# Continuing exposure to disadvantageous material and perceived economic factors on self-rated health in different life stages: fixed effects analyses with data from the German Socioeconomic Panel

**DOI:** 10.1186/s12889-024-21135-y

**Published:** 2025-02-04

**Authors:** Tobias Rähse, Matthias Richter, Anja Knöchelmann

**Affiliations:** 1 Institute of Medical Sociology, Interdisciplinary Centre for Health Sciences, Medical Faculty of the Martin Luther University Halle-Wittenberg, Halle (Saale), Saxony-Anhalt Germany; 2 Department Health and Sport Sciences, School of Medicine and Health, Technical University of Munich (TUM), Munich, Germany

**Keywords:** Life course epidemiology, Self-rated health, Disadvantageous factors, Accumulation, Adaptation, Healthy ageing

## Abstract

**Background:**

Life course epidemiology explores health disparities over time. The accumulation thesis thereby suggests an add-up of disadvantages, while the adaptation model assumes an adjustment to disadvantageous conditions. Examining the relevance of these accumulation and adaptation processes, the present study analyses continuing exposure to various material and perceived economic factors on self-rated health (SRH) across different life stages.

**Methods:**

All analyses are based on longitudinal data from the German Socio-Economic Panel (SOEP) from 1994 to 2017. Exposure variables, including loan burdens, housing status and quality (material factors) as well as financial and occupational worries, housing and income satisfaction (perceived economic factors), were analyzed dichotomously. Exposure duration was calculated as observed years in exposure for each of the factors, taking only continuous exposure years into account. The analyses were carried out separately for sex and life stages (emerging, early middle & later middle, late adulthood) using fixed effects models to adjust for time-varying covariates.

**Results:**

The analyses showed accumulation processes associated with housing status, financial worries and income satisfaction impacting SRH across most life stages. The effects of continuing exposure to occupational worries, housing satisfaction, housing quality, and loan burdens were more variable, indicating accumulation processes in certain life stages and sex-specific variations.

**Conclusions:**

While predominantly accumulation effects were found for certain factors, others showed more varied patterns. Future research should explore the mechanisms underlying these effects to develop well-timed measures that mitigate the negative health implications of continuing exposures to disadvantageous factors, emphasizing the importance of multiple exposures and later life health effects that may impede healthy ageing.

**Supplementary Information:**

The online version contains supplementary material available at 10.1186/s12889-024-21135-y.

## Background

Life course epidemiology (LCE) aims for a broad understanding of the onset and development of adverse health and health inequalities throughout the life course [1]. For this, different theoretical models and assumptions have been developed which are taking social, psychological and biological risk factors into account. One of the most well-proven models is the accumulation thesis, which states that (dis-)advantages longitudinally accumulate over the life course, resulting in worse health in later life. This has been shown, e.g., for self-rated health, health-related quality of life, psychiatric disorders and cardiovascular disease [2–6]. Traditionally, research on this model has measured accumulation by considering parental or individual educational, occupational or income position at childhood, young and late adulthood [7].

Another perspective focuses on identifying specific indicators and (mediating) factors which influence health throughout the life course, with particular attention to the time and timing of their occurrence [8]. Sensitive (or socially-critical) periods represent one way to explore this perspective, as they highlight how (dis-)advantageous factors act differently across different life stages [9]. Contrary to critical periods, such as childhood and adolescence, which are often linked to bio-physiological changes that can shape long-term health outcomes, sensitive periods do not necessarily coincide with these developments. Instead, they reflect windows during the life course where individuals may be particularly vulnerable to social, environmental, or psychological stressors, regardless of biological maturation [10]. Importantly, this perspective does not require exposure to be sustained across the entire life course. Rather, even temporary disadvantages during these periods can have long-lasting health effects.

The adaptation model takes a different approach and postulates that individuals get used to (dis-)advantageous situations. Health, life satisfaction and well-being drop at onset of a negative life event, increase afterwards and reach (almost) initial level, as measured before the occurrence of the event. This process is observed for a certain period of time rather than throughout the life course. Previous studies on life events regarding family, work and disability focused on time spans up to 7 years and the immediate effect on health and similar outcomes [11–16].

For some factors, such as unemployment, evidence for both accumulating as well as adaptation processes regarding health outcomes have been found [17–19]. While findings on accumulation processes indicate that persistent exposure to unemployment can lead to long-term health deterioration due to cumulative stressors such as the loss of financial resources and social contacts, research on adaptation processes suggests that individuals may build resilience through coping strategies like social support and learning from past experiences to mitigate negative health impacts. Therefore, the question arises whether they are opposing or rather complementary models. It might also be true that one model dominates in a certain period, while at a later point in life it becomes less prominent or even irrelevant.

Disadvantageous factors have been shown to play a crucial role in explaining health inequalities in cross-sectional research [20–25]. Studies on the long-term effects of these factors may shed a different light on the role of these exposures, as first results suggest that material factors are less important if measured repeatedly [26]. However, evidence is scarce and it is not clear whether continuing exposure to disadvantageous factors affect health more in terms of accumulation or adaptation and whether this differs with the studied exposures or the specific life stage in which it was experienced.

Investigating potential health impacts over a brief period could serve as a first step in examining accumulation and adaptation processes in a broader context.

Therefore, our aim is to study whether

i) continuing exposure to disadvantageous material and perceived economic factors affects health more in terms of accumulation or adaptation processes.
ii) the association between continuing exposure to disadvantageous material and perceived economic factors and health varies between different life stages.

## Methods

Analyses are based on longitudinal data, structured in long data format, from the German Socio-Economic-Panel (SOEP, version 34), an annual interdisciplinary study of private households in Germany. The SOEP provides detailed information about the living conditions and quality of life of the German population [27]. As respondents’ self-rated health (SRH) has been assessed since 1994, the data used derived from every SOEP wave from 1994 to 2017. Participants with less than two observations were excluded. The upper age limit was set at 75 years in order to avoid bias from selective mortality in older ages. The final sample included 248,886 observations from 30,904 women, averaging 12.59 observations per person and 223,709 observations from 28,421 men, with an average of 12.51 observations per person. The sample selection steps can also be drawn in more detail from the associated flowchart (Supplementary material 1).

### Outcome variable

Self-rated health (SRH) is generally considered to be strongly associated with morbidity and mortality, and thus with general health [28–31]. Due to its annual assessment, it allows to observe continual changes in respondents’ health status over time. SRH was initially measured using a five-point scale (very good (1) to poor (5)). We recoded this variable to a new range from 0 “poor” to 4 “very good” to ensure that higher values indicate better health status.

### Life stages

In line with other studies, we decided to analyze four life stages. Slightly adapted to the societal and cultural conditions in Germany, these cover the ages from 18 to 32 (emerging adulthood), 33 to 49 (early middle adulthood), 50 to 64 (later middle adulthood) and 65 to 75 years (late adulthood). During emerging adulthood, the prolonged transition to adulthood, individuals need to deal with several developmental tasks, such as entering the labor market and transitioning into marriage and parenthood, which can impact health and well-being [32]. Early middle adulthood is characterized by the challenge of balancing career progression with significant family responsibilities, particularly care for young- and school-aged children [33], while later middle adulthood represents a period where children typically gain independence, prompting individuals to refocus on personal and professional development, including reaching career peaks and preparing for retirement [33]. Finally, late adulthood encompasses the early years of retirement, often marked by lifestyle changes and shifting social networks [34].

### Material and perceived economic factors

All material and perceived economic factors were dichotomized, with 0 indicating non-exposure and 1 indicating exposure to the corresponding factor in the respective survey year.

#### Material factors

The variables for assessing material factors were selected according to their relevance and their ability to capture relevant dimensions of material well-being.

*Loan burdens* were selected to cover the financial strain on persons and households reflected in economic or even physical pressures they face from loan repayments [35]. To measure these burdens, observations for which respondents reported that they are repaying a loan and that these repayments are no problem were assigned to the code 0 (“no exposure to burdening loan repayment”). For observations in which participants stated loan repaying as a minor or major burden, the code 1 was assigned (“exposure to burdening loan repayment”).

*Housing status,* an indicator of economic stability and material security, influencing financial and psychological well-being [36, 37], was assessed based on the question of whether a participant is the owner of his residence. Non-ownership, including all forms of tenancy, was considered as exposure (code 1), while ownership, regardless whether apartment or house, was assigned to 0 (non-exposure).

*Housing quality* was measured by the degree of need for renovation of the own residence and including the values “good condition”, “partial renovation”, “full renovation” and “dilapidated”, as pivotal aspects of material deprivation [38]. The first category was classified as non-exposed (0), the following three were summarized as exposed to poor housing quality (code 1).

#### Perceived economic factors

We furthermore examined indicators that robustly capture individuals' subjective perception of their economic situation.

To reflect immediate and personal experiences of economic vulnerability, which have been shown to affect mental and physical health [39], *economic insecurities* were addressed using questions about financial and occupational worries with the response categories: “not concerned at all”, “somewhat concerned” and “very concerned”. The first category was assigned to code 0 for non-exposure, the latter two to code 1 to indicate exposure to financial or occupational insecurities, respectively. *Income and housing satisfaction* were measured using ten-point scales, with higher scores indicating higher satisfaction levels, proven predictors of life satisfaction and beneficial health [40]. For measuring exposure, we performed median splits [41]. All scores below median (7 for income, 8 for housing) were classified as below average satisfaction and assigned to the exposure categories (1). Values above and exactly at the median were categorized as (above) averagely satisfied and assigned to 0 (non-exposure).

Given these disadvantageous factors, it is important to also highlight the specific context of Germany [42]. Germany's robust social welfare system provides a wide range of protections, including nearly universal access to healthcare, unemployment benefits and public pensions, compared to less extensive welfare systems. Additionally, housing ownership may not carry the same weight in the German context due to the relative security and affordability of long-term renting. Moreover, student loan debt, that often contributes to long-term financial strain is far less prevalent in Germany. While these safety nets may buffer individuals from extreme material deprivation, they are not expected to eliminate the effects of continuing exposure to disadvantageous factors.

### Exposure duration

For each of the factors, observed years in exposure were added up, taking only continuous exposure years into account. If a participant was not exposed for one or more years or if no information was available, the exposure duration count was set to zero and restarted once a new exposure has been observed. Regarding accumulation, research on occupational hazards indicates that health effects may not manifest in the same way with interrupted exposure [43] and allostatic load tends to increase if exposure persists [44].

However, this approach also seems suitable for identifying adaptation processes. If exposure is intermittent, improvements in health status during non-exposure periods might merely reflect recovery due to the (temporary) removal of disadvantageous factors, rather than actual development of adaptation strategies. For example, research on stress adaptation highlights that sustained stress (rather than intermittent) provides a clearer indication of whether individuals develop effective coping mechanisms or not [45]. By focusing exclusively on continuous exposure, we therefore ensure that observed changes in SRH can be more reliably attributed to either accumulation of risk or to genuine adaptive strategies, thus facilitating a better comparison of these competing concepts.

Due to this approach, exposure durations which enclosed the entire observation period of up to 24 years occurred very rarely. For most factors no valid statements could be made beyond a continuous exposure period of 15 years, why we decided to limit the plotted associations between exposure duration and SRH to a maximum of 15 years.

### Control variables

To avoid bias from confounding variables, all performed models were adjusted for potential covariates such as components of social environment or socioeconomic position.

Regarding social environment, respondents’ family status (single, non-marital partnership, married) and the presence of a partner, as well as children (under the age of five years) in the household were considered. We further controlled for the region of residence (eastern vs. western Germany). Indicators of socioeconomic position were employment (full-time, part-time, none, pensioned, in education) and income position. The latter was measured using income quintiles, whereby the top quintile was associated with a high, the bottom one with a low and the middle three with a medium income position [46].

### Statistical analyses

The associations between disadvantageous factors and SRH are likely confounded by additional covariates, which cannot be easily controlled for (e.g., personality traits). To provide a clear and less biased view, fixed effects (FE) models were considered as the most suitable method for the statistical analyses. Since FE-models are implicitly controlling for any time-constant factor, only time-varying confounders remain to be observed [47]. Therefore, exposure towards all material and subjective economic factors as well as all covariates described in the previous section were treated as time-varying and included in the respective models accordingly. Average marginal effects (AME) and corresponding marginal effect plots were employed to provide information on the average change in the dependent variable (SRH) when the independent variables (disadvantageous factors) change from 0 to 1. The tabulated results of all FE-AME-models can be found in the Supplementary material 2 (App. Tables 1–14). All analyses were carried out separately for sex and life stages, to show potential differences in trajectories, given that sex-specific factors may influence health outcomes differently across the life course, as shown, e.g., for mental health aspects or allostatic load [48, 49]. Panel-robust standard errors were used in all estimated models, to adjust for clustering of observations within persons. All analyses were conducted using Stata version 18.

## Results

### Description

Descriptive statistics for the analysis are given in Table 1. Individuals in the final sample had a mean age of 45.0 years (females) and 45.4 years (males), and reported similar, moderate levels of SRH (women 2.41; men 2.49). The majority of the observations for both sexes were in early middle adulthood (women 38.26%; men 36.59%), followed by later middle (women 24.91%; men 26.19%), emerging (women 23.57%; men 23.38%) and late adulthood (women 13.26%; men 13.85%).

**Table 1:** Descriptive statistics of the sample over the entire observation period, by sex: German Socio-Economic Panel 1994–2017

	***Range***		**Women** ***Mean(SD)***	**Men** ***Mean(SD)***	**Women** ***%***	**Men** ***%***
Age	18	75	45.02 (15.16)	45.40 (15.37)		
Self-rated health	0	4	2.41 (0.95)	2.49 (0.93)		
Life stages						
Emerging adulthood	0	1			23.57	23.38
Early middle adulthood	0	1			38.26	36.59
Later middle adulthood	0	1			24.91	26.19
Late adulthood	0	1			13.26	13.85
Material and Perceived economic factors (years of exposure)						
Exposed to loan burden	0	12	1.55 (1.76)	1.52 (1.77)		
Exposed to non-ownership	0	24	2.98 (4.51)	2.78 (4.41)		
Exposed to bad housing quality	0	22	1.00 (2.25)	0.99 (2.25)		
Exposed to financial worries	0	24	3.52 (4.38)	3.25 (4.23)		
Exposed to occupational worries	0	24	1.44 (2.61)	1.70 (2.87)		
Exposed to housing dissatisfaction	0	24	0.97 (2.13)	1.00 (2.22)		
Exposed to income dissatisfaction	0	24	1.53 (2.86)	1.54 (2.90)		
Employment status						
Full-time employment	0	1			26.57	61.22
Part-time employment	0	1			28.17	5.52
Unemployed	0	1			21.92	8.76
Pensioned	0	1			16.71	17.26
In education	0	1			6.63	7.24
Income position						
Low income (income quintile 5)	0	1			21.88	18.40
Medium income (income quintiles 4–2)	0	1			59.33	60.06
High income (income quintile 1)	0	1			18.79	21.54
Social environment						
Children living in household	0	1			13.28	12.69
Partner living in household	0	1			25.42	27.68
Marital status						
Single (incl. divorced)	0	1			18.47	16.75
Married	0	1			63.96	66.87
Non-marital partnership	0	1			17.56	16.39
Residency						
East Germany	0	1			23.29	23.56
Observations			248,886	223,709		
Persons			30,904	28,421		

Average exposure durations to the disadvantageous factors appeared largely similar for both sexes. While men experienced slightly longer exposure to factors such as occupational worries (women = 1.44 years; men = 1.70 years) as well as housing (women = 0.97; men = 1.00) and income dissatisfaction (women = 1.53; men = 1.54), women experienced longer exposure durations regarding loan burden (women = 1.55; men = 1.52), non-ownership (women = 2.98; men = 2.78), bad housing quality (women = 1.00; men = 0.99) and financial worries (women = 3.52; men = 3.25).

### Investigating continuing exposures on SRH: FE-models

#### Loan burden

**Fig. 1:**
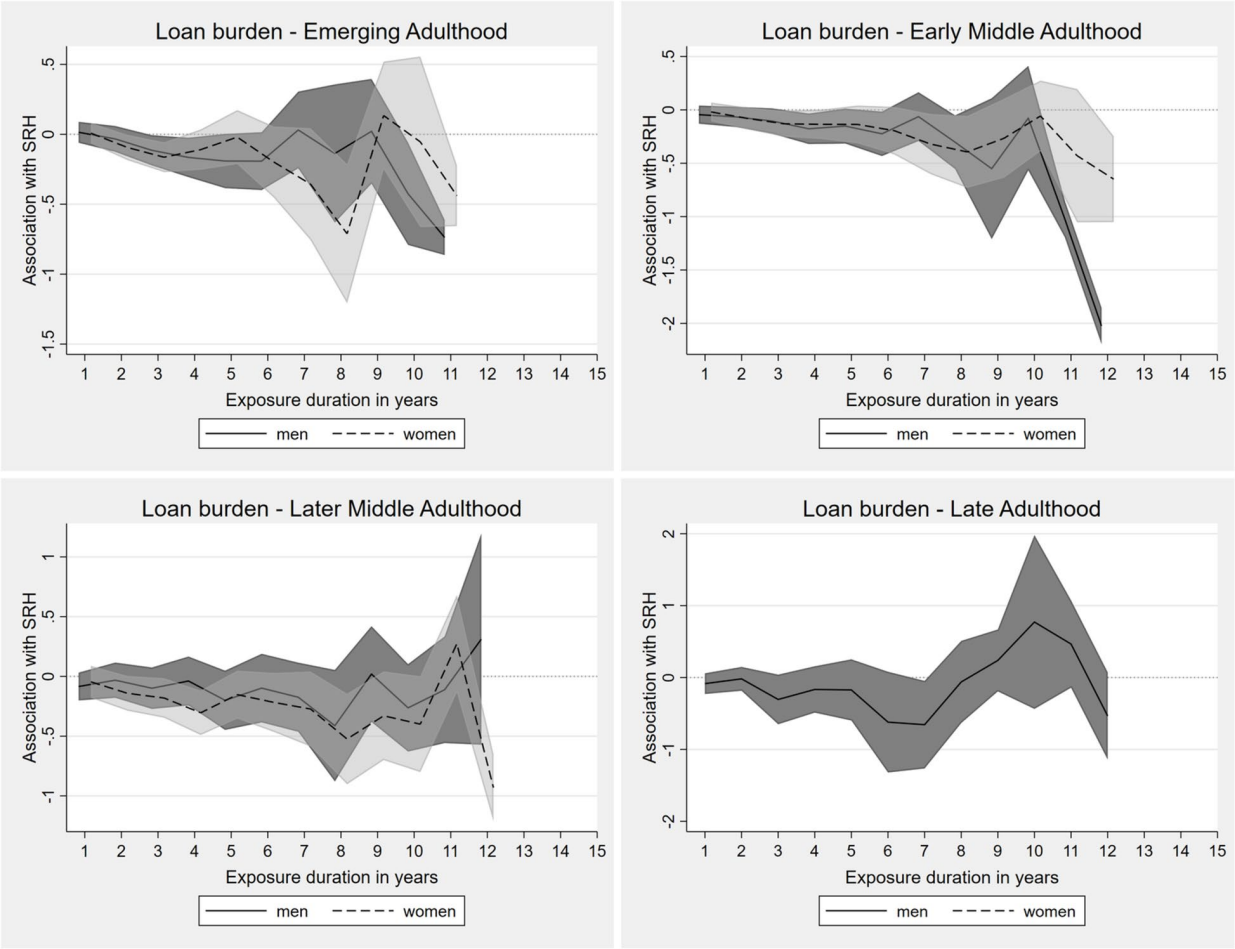
Fixed-effects regression for impact of continuing exposure to loan burden on SRH in different life stages (presented as AMEs). Adjusted for employment status, income position, marital status, social environment, residency

Negative associations of exposure to loan burdens with SRH were already evident in emerging adulthood (Fig. 1). Initially, the effect was rather small for both sexes. For men, a decline in SRH could be observed until 6 years of exposure with an additional steep decline after 9 years of continuing exposure to loan burdens. For women, a negative effect could be seen after more than 5 years of exposure duration, followed by a steep incline after 8 years and a recurrent negative impact after 9 years.

In early middle adulthood, significant associations were found only for the longest exposure durations among men. For women a quite consistent, but rather modest, trend of accumulation of disadvantages regarding their SRH due to loan burdens was observed. Neither for men nor for women, hardly any reliable effects indicating accumulation and/or adaptation processes could be found in later middle adulthood. Similar results were found for men in late adulthood, whereas insufficient data was available for women in this life stage to estimate valid margins.

#### Housing status

**Fig. 2:**
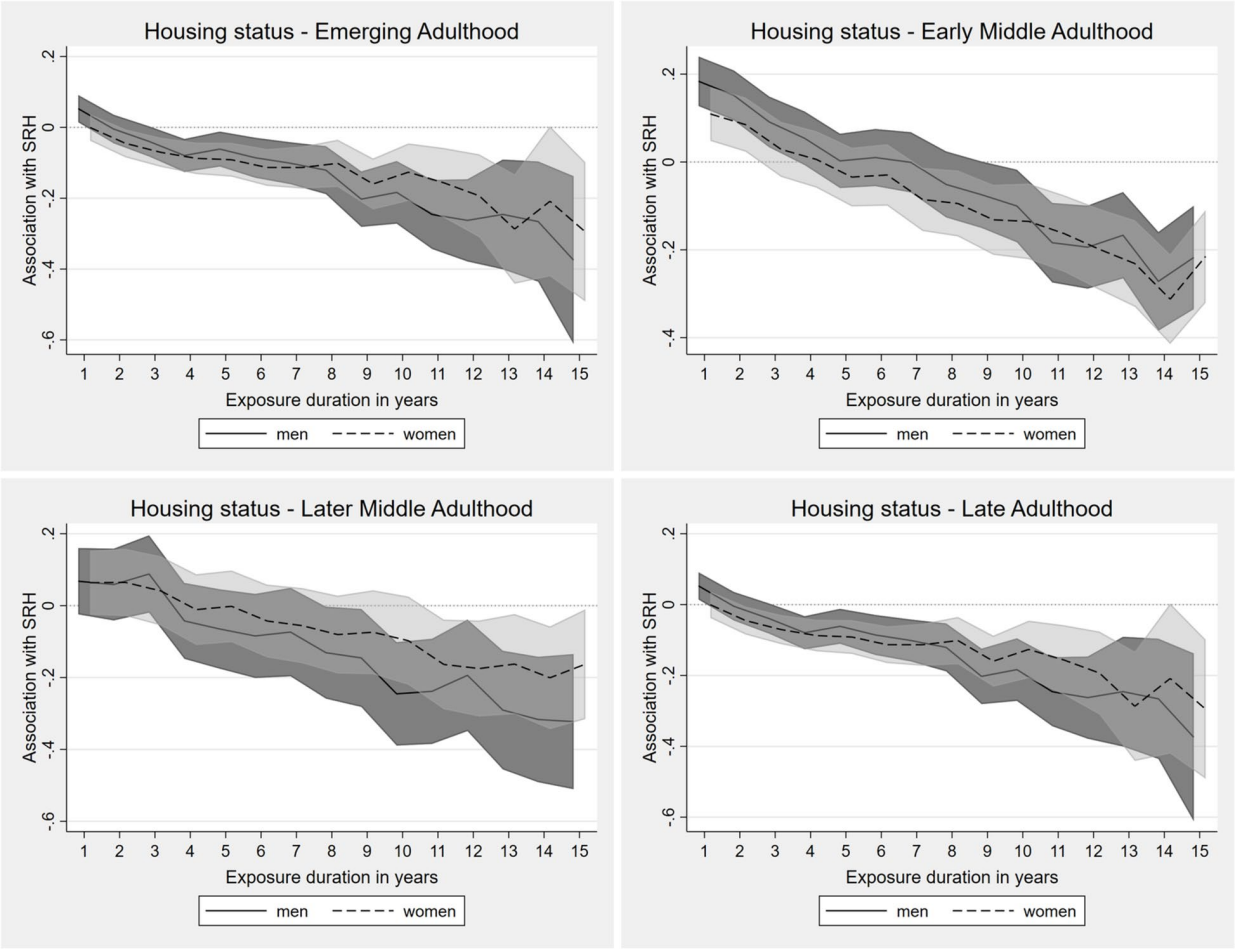
Fixed-effects regression for impact of continuing exposure to housing non-ownership on SRH in different life stages (presented as AMEs). Adjusted for employment status, income position, marital status, social environment, residency

Initially, non-ownership had a positive effect on SRH, whereby this correlation is particularly clear for early and later middle adulthood individuals and lasting up to an exposure period of 4 years (Fig. 2). Afterwards, the absence of home ownership was negatively associated with SRH in all life stages. This significant and highly consistent negative association became stronger with increasing exposure, suggesting accumulation processes. For women, these effects were already significant after a shorter exposure period and partly even more pronounced than for men, except in later middle adulthood.

#### Housing quality

**Fig. 3:**
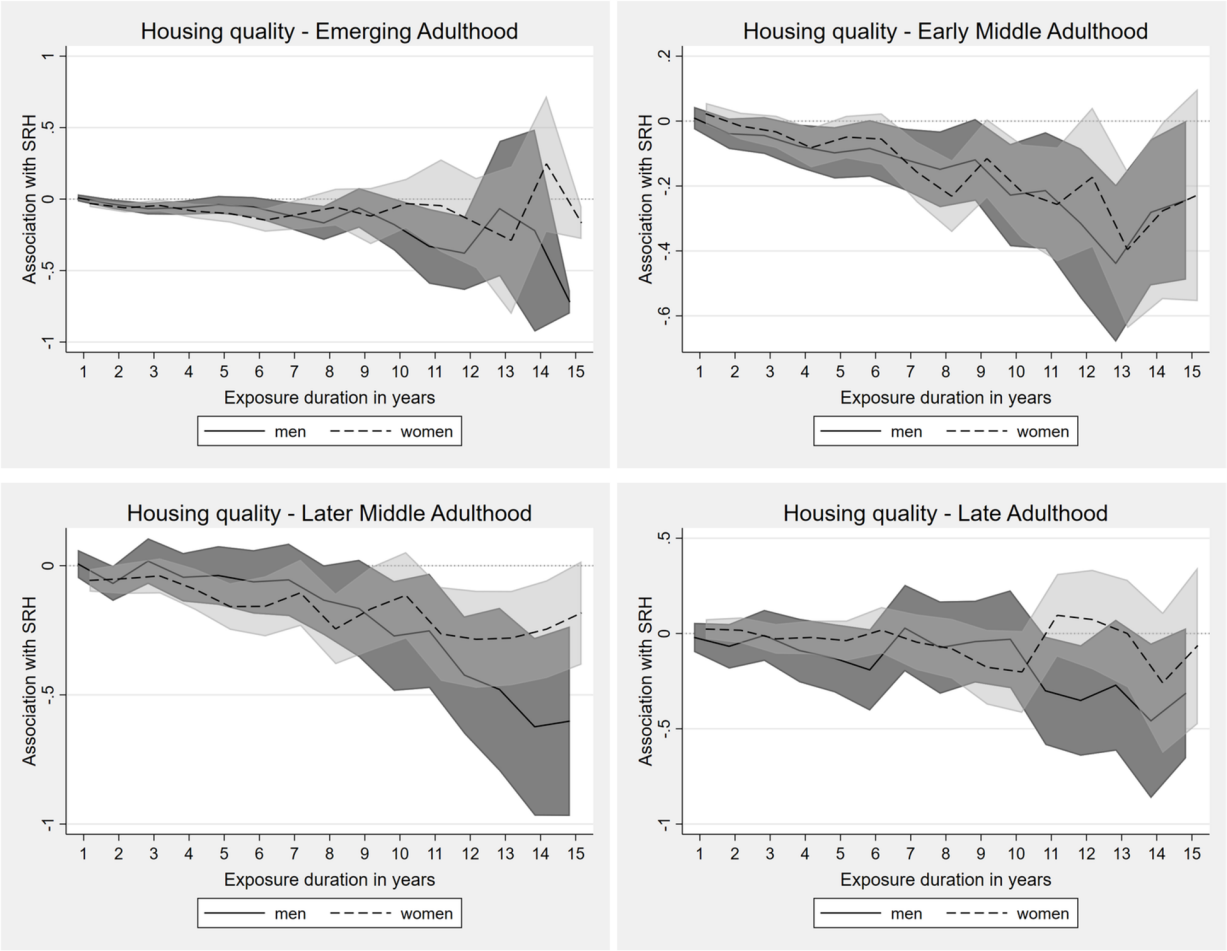
Fixed-effects regression for impact of continuing exposure to bad housing quality on SRH in different life stages (presented as AMEs). Adjusted for employment status, income position, marital status, social environment, residency

In emerging and late adulthood, exposure to poor housing quality seemed to be less relevant for SRH, whereas in early and later middle adulthood, stronger associations emerged (Fig. 3). Men appeared to be more affected in terms of accumulation of disadvantages, which was reflected in decreasing SRH, especially for continuing exposure for 10 or more years. Among women, similar trends were found in these two life stages, although not as prominent.

#### Economic insecurities

**Fig. 4:**
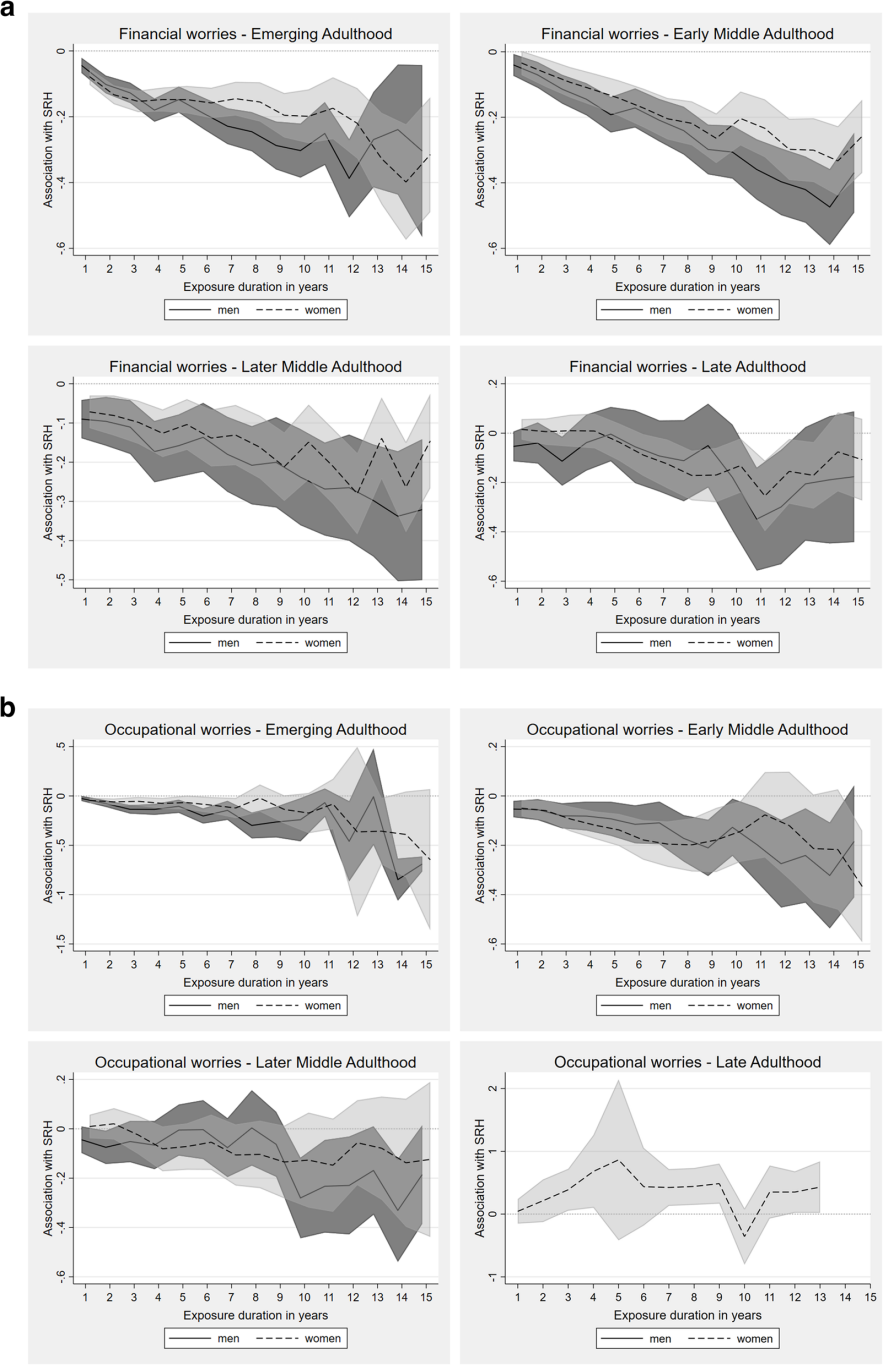
**a** Fixed-effects regression for impact of continuing exposure to financial worries on SRH in different life stages (presented as AMEs). Adjusted for employment status, income position, marital status, social environment, residency. **b** Fixed-effects regression for impact of continuing exposure to occupational worries on SRH in different life stages (presented as AMEs). Adjusted for employment status, income position, marital status, social environment, residency

Regarding economic insecurity, substantial and persistent accumulation effects were evident regarding financial worries, which increased with longer exposure duration (Fig. 4a). The process was less pronounced in late adulthood, especially for men. The exposure to financial worries was strongly negatively associated with SRH, with longer exposure being associated with greater deterioration in health. These associations were statistically significant for men and women over the entire observation periods in the first three life stages, most prominent for early middle adulthood.

Considering occupational worries, no valid statements about late adulthood could be made, especially for men where no margins were estimable at all, since the majority of individuals in this life stage were already retired (Fig. 4b). In emerging adulthood, however, adverse effects of long-term exposure to occupational worries on SRH were seen for men, but merely so for women at this stage. In early middle adulthood a growing decline of SRH with increasing exposure for durations up to seven years could be seen. For men, this effect was also observed for even longer continuing exposure (accumulation). In women, the SRH scores tended to return to the initial level with increasing exposure, suggesting, if at all, an adaptation process, followed by a recurrent decline in SRH after 11 years of exposure. In later middle adulthood, the associations were less strong overall and the sex differences less prominent. Yet, a negative breaking point starting at an exposure duration of ten years, after which SRH levels dropped significantly with further exposure was observed, but only in men.

#### Housing and income satisfaction

**Fig. 5:**
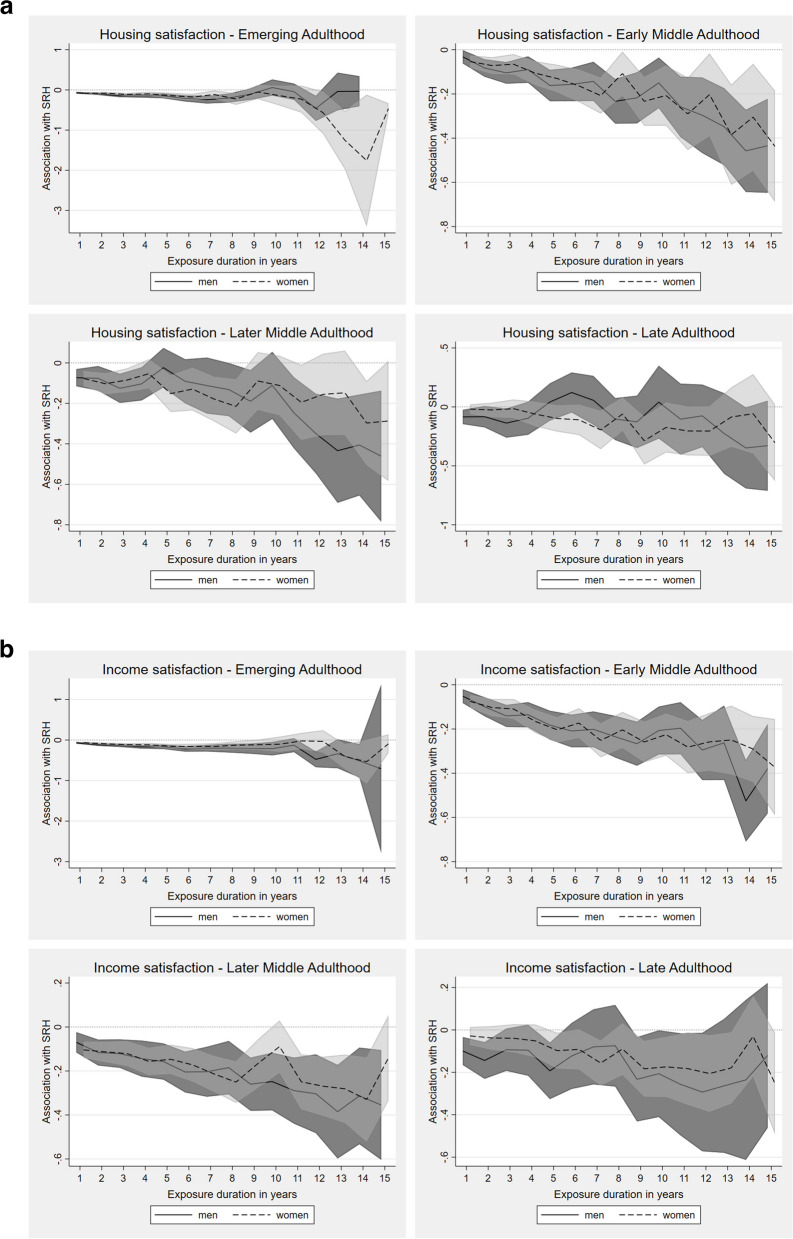
**a** Fixed-effects regression for impact of continuing exposure to below-average housing satisfaction on SRH in different life stages (presented as AMEs). Adjusted for employment status, income position, marital status, social environment, residency. **b** Fixed-effects regression for impact of continuing exposure to below-average income satisfaction on SRH in different life stages (presented as AMEs). Adjusted for employment status, income position, marital status, social environment, residency

In emerging adulthood, below-average housing satisfaction initially affected men's and women's SRH similarly, with significant, modestly changing SRH-trajectories for exposure durations of up to ten years (Fig. 5a). After that, men's SRH scores became insignificant, while women's SRH scores began to decline (sharply) with each additional exposure year, indicating accumulation.

In early middle adulthood, exposure to below-average housing satisfaction was associated with a consistently significant decrease of SRH for both sexes, which became stronger with increasing exposure duration, again suggesting the presence of accumulation processes. A similar development was seen in later middle adulthood, with associations being more pronounced in men, especially at longer exposure durations. For women, the same overall trend was evident, but only significant for exposure durations up to eight years, with inconsistencies thereafter. In late adulthood, effects were highly variable, indicating neither accumulation nor adaptation.

Similar to housing satisfaction, negative associations between below-average income satisfaction and SRH were already found in emerging adulthood for both sexes, albeit again rather modest in size (Fig. 5b). Continuing exposures in early and later middle adulthood were strongly associated with substantial declines in SRH, indicating accumulation, despite an intermediate steep incline between eight and ten years of exposure for women in later middle adulthood. In late adulthood, these associations were less strong and barely substantial again, especially among men.

## Discussion

Accumulation processes were seen for the majority of material and perceived economic factors and SRH. This association was particularly strong for housing status and financial worries. Contrary to Groeniger et al., we did not see a reduced relevance of these material factors when measured longitudinally [26]. This discrepancy might be attributed to the differing outcomes considered (SRH vs. mortality), as Groeniger et al. suggest that material disadvantages tend to exert more indirect effects on mortality, which may initially manifest in poorer (subjective) health [26]. Regarding life stages, early and later middle adulthood showed the most noticeable accumulation processes, with continuing exposure to material and perceived economic factors significantly impacting SRH. This continuing disadvantage could exacerbate health inequalities as individuals, especially during these periods, are facing societal pressures to meet professional and economic goals. These results are in line with findings from previous studies which showed the importance of accumulation processes for health inequalities, focusing on indicators of socioeconomic position [2–6]. In contrast, continuing exposure to material and perceived economic factors seems to be less relevant for self-rated health in emerging and late adulthood. Our study extends the existing research on life course epidemiology as well as on material and perceived economic factors, as we were able to show that not only income or education are important for accumulation of health disadvantages, but that perceived economic and material factors are also relevant in a longitudinal perspective.

*Housing status* emerged as a crucial material factor influencing SRH across all life stages. The absence of housing ownership exhibited a consistent negative association with SRH after an initial short-term positive effect. Housing status might act as a proxy for various dimensions of well-being, such as quality of life or as a status symbol, reflecting a person’s achievements and social standing within a community [36, 37]. Home ownership can be perceived as a psychological anchor, fostering a feeling of security and stability [50], as homeowners are less likely to live in deprived neighborhoods than renters and/or more invested in improving neighborhood conditions [51].

Furthermore, the pursuit of *home ownership* is likely to be seen as a life goal that individuals aspire to and may work towards throughout their life course [52]. The ongoing inability of reaching this goal might be burdensome and could explain the negative effect on SRH. Along with the absence of a cushioning effect of ownership as potential sales asset and thus retirement provision [53], this may contribute to the enduring importance of home ownership into late adulthood, where not achieving this goal continues to be important for SRH. The short-term positive effects of non-ownership may stem from the initial burdens of starting owners due to planning, financial and construction necessities.

For perceived economic factors, *income* and *housing satisfaction* seemed most relevant. The subjective perceptions of satisfaction persists even when controlling for objective factors such as income or occupational position, in line with prior research [40, 54]. Social comparison with others seems to play a pivotal role for explaining these results, especially for income satisfaction [55]. Persons feeling inferior in social comparison may tend to avoid such comparisons and become socially isolated [56]. Regarding housing satisfaction, it has been evidenced that persons living in deprived neighborhoods suffer from a lack of crucial resources such as healthy lifestyles and food options as well as social support which, alongside social isolation, can negatively impact SRH [57, 58].

In general, the impact of continuing exposure to material and perceived economic factors was more pronounced in early and later middle adulthood. This might be explained by various personal and societal expectations and norms which are dictating certain professional, economic and familial goals that need be achieved within these stages to be considered ‘successful’ [33, 59]. The feeling of not having achieved these goals fully or in time can be perceived as a burden, exacerbating stress on the individuals, which might result in worse health. The explanation for heterogenous results regarding emerging adulthood can also be found in the characteristics of this life stage. While society has clearly defined requirements regarding achievements in early and later middle adulthood, they are less important in emerging adulthood. Here, persons are expected to follow their individual paths to lay the foundation for a future career and an organized family life, thereby establishing the groundwork for the requirements of early and later middle adulthood [60]. It is predictable and inevitable that this is accompanied by lower income and lower housing quality. Hence, it can be assumed that these factors have a less significant impact on immediate health, as dissatisfaction with income or living conditions, or the burden of debt, might be viewed as temporary, with the prospect of improvement in future life mitigating negative health effects. Regarding late adulthood, the diminished relevance of continuing exposure to disadvantageous factors for self-rated health in this life stage may be explained by the reduced effectiveness of cultural resources, including material and (perceived) economic factors, due to decreasing biological plasticity associated with higher age [61]. Moreover, research suggests that subjective health perceptions tend to become less reliable or relevant compared to objective health status in late adulthood, as individuals in this stage report greater life satisfaction, which can result in contradictory effects, with some older adults reporting improvements in subjective well-being over time, even if their objective health declines [62]. However, delayed effects of continuing exposures to past disadvantages may still play a role in shaping older adults’ health status and perception but may not have been fully captured due to limitations in sample size, as discussed in the following limitations section.

Sex differences were only seen for some of the studied factors and mainly in early to later middle adulthood with longer times in disadvantages, with men showing worse health than women. This might be due to the still existing typical role expectations, with men being more likely to be expected to fulfill job and income related obligations, whereas women are oftentimes responsible to provide a representable home [63]. This reflects in our results where women only have worse health than men regarding the need for renovation.

### Strengths and limitations

The SOEP is representative of the German population with a high number of respondents and covers a long observation period. The substantial number of participants allowed us to explore the relationship between perceived economic and material factors and SRH across various life stages for men and women respectively. Due to the continuous and consistent collection of the data we were able to address the importance of these factors longitudinally. We used Fixed-effects regression to analyze the impact of material and perceived economic factors on SRH. Therefore, we were able to account for all unobserved time-invariant factors, which might influence the studied associations, such as personality traits.

We decided not to add up the observed years of exposure over the entire life course, but to take only continuous exposure years into account, as health related recovery might occur in non-exposed years. While allowing a more precise examination of continuing exposures, this approach may underestimate that a relapse in exposure could pose an additional weight on the disadvantage and could lead to persons being considered multiple times, if after (at least) one year of non-exposure another exposure period was observed for the same participant.

Due to the upper age restrictions, late adulthood has the lowest number of cases. Consequently, the observed effects for this stage are modest in size and less substantial – considering people over this age limit could have led to different or more robust results. However, we considered imprecision due to age-associated multimorbidity and mortality to be a major confounding factor and therefore decided to use an upper age limit for the inclusion of observations.

While our methodical approach of focusing exclusively on continuing exposure aims to compare the presence and extent of accumulation and adaptation processes under equal conditions, our analyses primarily provide insights into the accumulation of risk and may not fully capture the processes of adaptation. As a result, alternative methods, e.g., as used by Clark et al. or Luhmann et al. might be more suitable for comprehensively identifying adaptation processes [13, 15].

Moreover, the non-availability of certain material, perceived and behavioral indicators, due to inconsistencies and data gaps in the SOEP dataset, along with the lack of consideration for the potentially enhancing effects of multiple exposures, may limit the comprehensiveness of our analyses. The same applies to potential bias introduced by the possibility of control variables succeeding exposure variables in some individual cases.

## Conclusion

Our findings shed light on the role of material and perceived economic factors for SRH from a longitudinal perspective, suggesting that health is more affected by continuing exposure to disadvantages during certain life stages, emphasizing that the timing and persistence of these factors can profoundly influence health outcomes. This slightly points towards the existence of sensitive periods marked by timing-dependent vulnerability also besides the biophysical dimension of critical periods. For some of the analyzed factors, most noticeable for housing status, financial worries and income satisfaction, continuing disadvantage was associated with a deterioration in health, indicating accumulation processes, while no reliable effects were discernible supporting the adaptation model. This has been found for almost all life stages, most pronounced in early and later middle adulthood. For other factors, such as occupational worries, housing satisfaction and quality or loan burdens, paths are more heterogeneous. However, they also primarily tend towards accumulation processes or have no impact on SRH at all. We did not find patterns of sex-related differences other than that for some factors steeper accumulation processes were shown for men in early and later middle adulthood.

Future research should delve deeper into the mechanisms driving these patterns, particularly focusing on how prolonged exposure to disadvantageous factors over the life course influences health outcomes, to offer more effective strategies for reducing health disparities. Building on our results, potential avenues for such interventions could be, for instance, policies aiming at improving housing conditions, reducing income inequality or strengthening supportive communities, to target deprived neighborhoods and social isolation, which appear to be key mechanisms behind the most severe disadvantageous factors of housing and income satisfaction as well as financial worries.

While the present study focuses on quite immediate health effects, investigating long-term effects seems also crucial because as risk factors might coincide and interact with each other in the long-term, their effects could reinforce, exacerbate health issues and thus significantly reduce the likelihood of healthy aging. By understanding these long-term processes, we can better identify not only avenues but also promising time windows for effective interventions that could mitigate such adverse health and aging implications.

## Supplementary Information

Supplementary Material 1.

Supplementary Material 2.

## Data Availability

The raw data contained in this manuscript are not openly available due to data protection restrictions of the German Institute for Economic Research (DIW), which collects and provides the SOEP data. SOEP data are available free of charge for scientific use from the DIW at https://www.diw.de/en/diw_01.c.601584.en/data_access.html after signing a data distribution contract. Data transformation files and codes are available from the corresponding author upon reasonable request.

## Abbreviations

LCE: Life Course Epidemiology
SRH: Self-Rated Health
SOEP: Socio-Economic Panel
SD: Standard Deviation
FE: Fixed Effects
AME: Average Marginal Effects
