# Supplementary material for: Continuing exposure to disadvantageous material and perceived economic factors on self-rated health in different life stages: fixed effects analyses with data from the German Socioeconomic Panel
Source: BMC Public Health. 2025 Feb 4;25:446. doi: 10.1186/s12889-024-21135-y (PMC11792312; doi:10.1186/s12889-024-21135-y)

Suppl. File 1 Flowchart illustrating the steps of sample selection with inclusion and exclusion criteria

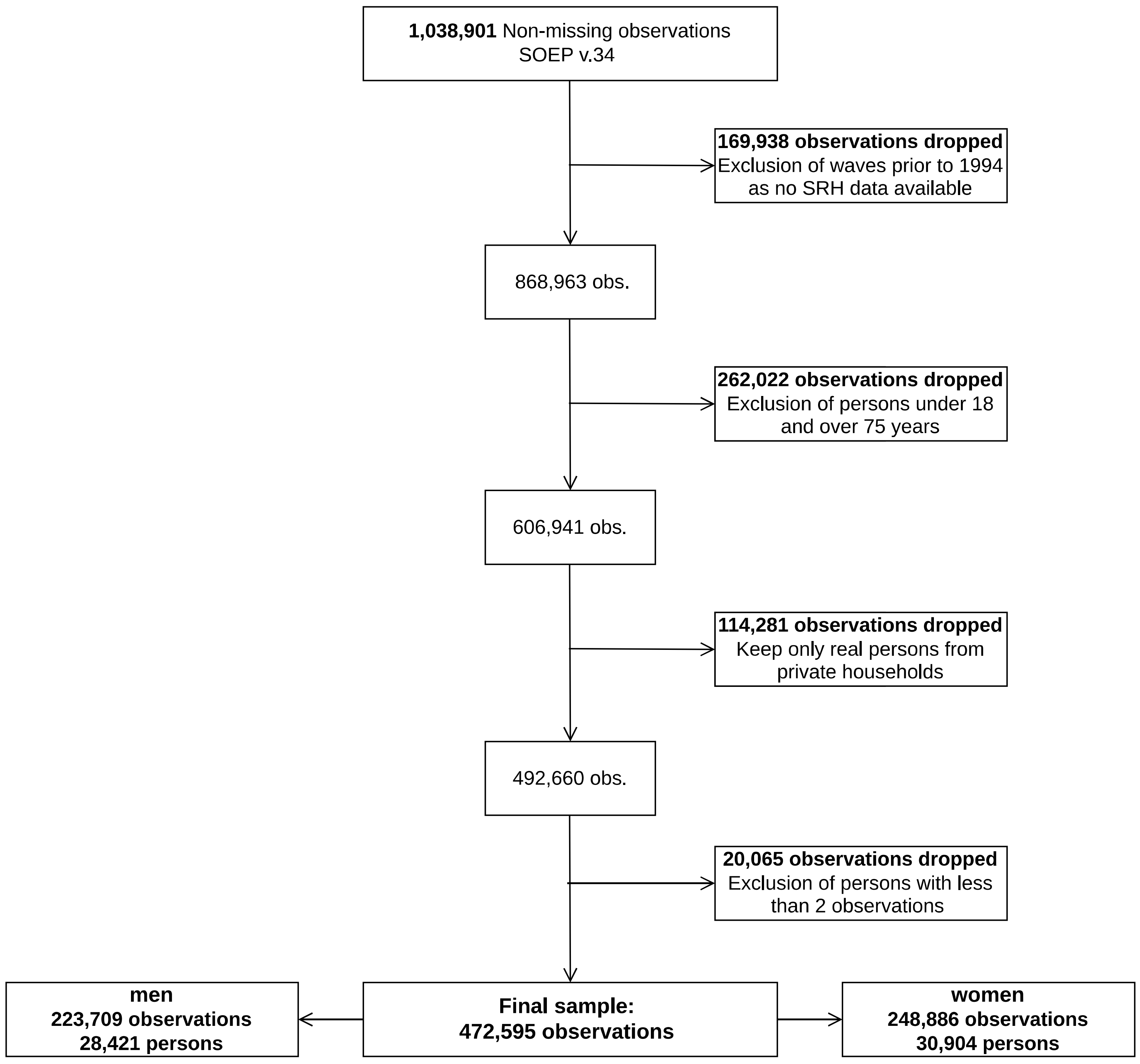

Supplement: Supplementary file 1 — Supplementary Material 1. [file 12889_2024_21135_MOESM1_ESM.pdf]
