# Supplementary material for: Continuing exposure to disadvantageous material and perceived economic factors on self-rated health in different life stages: fixed effects analyses with data from the German Socioeconomic Panel
Source: BMC Public Health. 2025 Feb 4;25:446. doi: 10.1186/s12889-024-21135-y (PMC11792312; doi:10.1186/s12889-024-21135-y)
Supplement: Supplementary file 2 — Supplementary Material 2. [file 12889_2024_21135_MOESM2_ESM.docx]

**App. Table 1** Fixed-effects regressions for impact of continuing exposure to loan burden on SRH in different life stages (regression outputs)

|  | Women | | | | Men | | | |
| --- | --- | --- | --- | --- | --- | --- | --- | --- |
| Impact on Self-rated health | Emerging Adulthood | Early Middle Adulthood | Later Middle Adulthood | Late Adulthood | Emerging Adulthood | Early Middle Adulthood | Later Middle Adulthood | Late Adulthood |
|  |  |  |  |  |  |  |  |  |
| No Exposure | Ref. | Ref. | Ref. | Ref. | Ref. | Ref. | Ref. | Ref. |
| Exposure Duration: 1 year | 0.008 | -0.019 | -0.046 | 0.139 | 0.014 | -0.044 | -0.084 | -0.077 |
|  | (0.038) | (0.045) | (0.068) | (0.168) | (0.038) | (0.043) | (0.059) | (0.103) |
| Exposure Duration: 2 years | -0.094** | -0.080 | -0.141* | 0.066 | -0.033 | -0.065 | -0.032 | 0.032 |
|  | (0.046) | (0.052) | (0.074) | (0.191) | (0.047) | (0.048) | (0.075) | (0.128) |
| Exposure Duration: 3 years | -0.164*** | -0.130** | -0.181** | -0.060 | -0.115** | -0.104* | -0.099 | -0.309 |
|  | (0.055) | (0.062) | (0.084) | (0.191) | (0.055) | (0.060) | (0.088) | (0.251) |
| Exposure Duration: 4 years | -0.109 | -0.136* | -0.305*** | 0.164 | -0.165** | -0.177** | -0.038 | -0.112 |
|  | (0.074) | (0.070) | (0.095) | (0.209) | (0.072) | (0.073) | (0.104) | (0.222) |
| Exposure Duration: 5 years | -0.020 | -0.136 | -0.152 | 0.167 | -0.191* | -0.152* | -0.200 | -0.014 |
|  | (0.099) | (0.090) | (0.102) | (0.225) | (0.099) | (0.083) | (0.126) | (0.241) |
| Exposure Duration: 6 years | -0.199 | -0.194* | -0.217* | -0.077 | -0.192* | -0.224** | -0.098 | -0.363 |
|  | (0.130) | (0.113) | (0.126) | (0.196) | (0.105) | (0.106) | (0.146) | (0.433) |
| Exposure Duration: 7 years | -0.356* | -0.320** | -0.273* | 0.268 | 0.031 | -0.063 | -0.174 | -0.410 |
|  | (0.204) | (0.144) | (0.160) | (0.199) | (0.140) | (0.117) | (0.148) | (0.277) |
| Exposure Duration: 8 years | -0.711*** | -0.394** | -0.524*** | 0.662 | -0.137 | -0.303** | -0.413* | -0.520 |
|  | (0.254) | (0.172) | (0.193) | (0.606) | (0.252) | (0.128) | (0.238) | (0.329) |
| Exposure Duration: 9 years | 0.134 | -0.266 | -0.329* | - | 0.021 | -0.551 | 0.019 | -0.347 |
|  | (0.197) | (0.189) | (0.189) | - | (0.191) | (0.336) | (0.204) | (0.261) |
| Exposure Duration: 10 years | -0.054 | -0.057 | -0.400* | - | -0.425** | -0.077 | -0.263 | -0.347 |
|  | (0.311) | (0.169) | (0.205) | - | (0.187) | (0.249) | (0.187) | (0.261) |
| Exposure Duration: 11 years | -0.439*** | -0.429 | 0.273 | 0 | -0.738*** | -1.024*** | -0.112 | -0.361 |
|  | (0.111) | (0.319) | (0.208) | (omitted) | (0.064) | (0.087) | (0.228) | (0.430) |
| Exposure Duration: 12 years | - | -0.649*** | -0.931*** | 0 | - | -2.024*** | 0.311 | -1.361*** |
|  | - | (0.205) | (0.144) | (omitted) | - | (0.087) | (0.450) | (0.430) |
|  |  |  |  |  |  |  |  |  |
| Full-time employment | Ref. | Ref. | Ref. | Ref. | Ref. | Ref. | Ref. | Ref. |
| Part-time employment | 0.093 | -0.046 | -0.060 | 0.571* | 0.104* | -0.083 | -0.061 | 0.211 |
|  | (0.056) | (0.055) | (0.088) | (0.332) | (0.059) | (0.141) | (0.127) | (0.290) |
| No employment | 0.248*** | -0.075 | -0.183 | 0 | 0.047 | -0.138 | -0.227* | -0.267 |
|  | (0.066) | (0.091) | (0.156) | (omitted) | (0.062) | (0.089) | (0.129) | (0.326) |
| Pensioners | 0.969*** | -0.419 | -0.089 | 0.486 | 1.294*** | -0.437** | 0.356* | -0.553** |
|  | (0.258) | (0.260) | (0.132) | (0.320) | (0.344) | (0.188) | (0.195) | (0.238) |
| In education | 0.083 | -0.254 | 0.310 | 0 | 0.031 | 0.201 | 0.125 | - |
|  | (0.051) | (0.204) | (0.397) | (omitted) | (0.044) | (0.266) | (0.347) | - |
|  |  |  |  |  |  |  |  |  |
| Low income position | -0.048 | 0.047 | -0.097 | -0.118 | -0.044 | 0.054 | -0.099 | 0.117 |
|  | (0.051) | (0.052) | (0.084) | (0.208) | (0.053) | (0.077) | (0.120) | (0.265) |
| Medium income position | Ref. | Ref. | Ref. | Ref. | Ref. | Ref. | Ref. | Ref. |
| High income position | 0.016 | -0.015 | 0.069 | -0.440** | 0.010 | -0.037 | 0.053 | 0.433* |
|  | (0.058) | (0.072) | (0.089) | (0.222) | (0.050) | (0.062) | (0.084) | (0.261) |
|  |  |  |  |  |  |  |  |  |
| Single (incl. divorced) | Ref. | Ref. | Ref. | Ref. | Ref. | Ref. | Ref. | Ref. |
| Married | -0.033 | 0.148 | 0.124 | -0.096 | -0.276 | 0.016 | 0.204 | 0.296 |
|  | (0.177) | (0.099) | (0.132) | (0.135) | (0.345) | (0.085) | (0.146) | (0.327) |
| Non-marital partnership | 0.025 | 0.038 | 0.121 | 0.009 | -0.052 | 0.071 | 0.034 | 0.121 |
|  | (0.048) | (0.059) | (0.078) | (0.130) | (0.039) | (0.067) | (0.092) | (0.159) |
|  |  |  |  |  |  |  |  |  |
| No partner in household | Ref. | Ref. | Ref. | Ref. | Ref. | Ref. | Ref. | Ref. |
| Partner living in household | 0.009 | -0.058 | -0.410*** | 0.004 | 0.068 | -0.079 | 0.016 | -0.736* |
|  | (0.065) | (0.075) | (0.159) | (0.142) | (0.067) | (0.066) | (0.088) | (0.398) |
|  |  |  |  |  |  |  |  |  |
| No children<5 in household | Ref. | Ref. | Ref. | Ref. | Ref. | Ref. | Ref. | Ref. |
| Household with children<5 | -0.029 | -0.071 | 0.215 | 0 | -0.042 | 0.065 | 0.413 | 0 |
|  | (0.063) | (0.073) | (0.215) | (omitted) | (0.086) | (0.074) | (0.292) | (omitted) |
|  |  |  |  |  |  |  |  |  |
| Western Germany | Ref. | Ref. | Ref. | Ref. | Ref. | Ref. | Ref. | Ref. |
| Eastern Germany | -0.023 | 0.146 | -0.167 | 0 | 0.236 | 0.097 | -0.058 | -0.113 |
|  | (0.128) | (0.173) | (0.127) | (omitted) | (0.153) | (0.350) | (0.062) | (0.227) |
|  |  |  |  |  |  |  |  |  |
| Constant | 2.656*** | 2.385*** | 2.283*** | 1.327*** | 2.848*** | 2.527*** | 2.092*** | 2.368*** |
|  | (0.065) | (0.076) | (0.095) | (0.341) | (0.063) | (0.121) | (0.092) | (0.319) |
|  |  |  |  |  |  |  |  |  |
| Observations | 4,674 | 4,141 | 1,954 | 414 | 4,781 | 3,084 | 1,479 | 286 |

German Socio-Economic Panel 1994-2017; Significance levels are *p<0.05, **p<0.01 and ***p<0.001; Corresponds to Figure 1

Shown are unstandardized beta coefficients (β) and panel robust standard errors (SE)

**App. Table 2** Predictive margins on changes in SRH for each year of continuing exposure towards loan burden in different life stages

|  | Women | | | | Men | | | |
| --- | --- | --- | --- | --- | --- | --- | --- | --- |
| Predictive margins | Emerging Adulthood | Early Middle Adulthood | Later Middle Adulthood | Late Adulthood | Emerging Adulthood | Early Middle Adulthood | Later Middle Adulthood | Late Adulthood |
|  |  |  |  |  |  |  |  |  |
| No exposure | Ref. | Ref. | Ref. | Ref. | Ref. | Ref. | Ref. | Ref. |
| Exposure dur.: 1 yr | 0.008 | -0.019 | -0.046 | not estimable | 0.014 | -0.044 | -0.084 | -0.077 |
|  | (-0.066 - 0.082) | (-0.107 - 0.068) | (-0.180 - 0.088) |  | (-0.061 - 0.089) | (-0.129 - 0.041) | (-0.201 - 0.032) | (-0.279 - 0.125) |
| Exposure dur.: 2 yrs | -0.094** | -0.080 | -0.141* | not estimable | -0.033 | -0.065 | -0.032 | 0.032 |
|  | (-0.185 - -0.004) | (-0.182 - 0.022) | (-0.287 - 0.004) |  | (-0.125 - 0.059) | (-0.160 - 0.029) | (-0.179 - 0.116) | (-0.219 - 0.282) |
| Exposure dur.: 3 yrs | -0.164*** | -0.130** | -0.181** | not estimable | -0.115** | -0.104* | -0.099 | -0.309 |
|  | (-0.271 - -0.057) | (-0.252 - -0.008) | (-0.346 - -0.015) |  | (-0.223 - -0.007) | (-0.223 - 0.014) | (-0.272 - 0.074) | (-0.800 - 0.182) |
| Exposure dur.: 4 yrs | -0.109 | -0.136* | -0.305*** | not estimable | -0.165** | -0.177** | -0.038 | -0.112 |
|  | (-0.254 - 0.035) | (-0.273 - 0.001) | (-0.491 - -0.119) |  | (-0.306 - -0.025) | (-0.320 - -0.034) | (-0.243 - 0.166) | (-0.548 - 0.324) |
| Exposure dur.: 5 yrs | -0.020 | -0.136 | -0.152 | not estimable | -0.191* | -0.152* | -0.200 | -0.014 |
|  | (-0.213 - 0.173) | (-0.314 - 0.041) | (-0.353 - 0.048) |  | (-0.385 - 0.003) | (-0.315 - 0.012) | (-0.448 - 0.048) | (-0.486 - 0.458) |
| Exposure dur.: 6 yrs | -0.199 | -0.194* | -0.217* | not estimable | -0.192* | -0.224** | -0.098 | -0.363 |
|  | (-0.453 - 0.056) | (-0.417 - 0.028) | (-0.464 - 0.030) |  | (-0.399 - 0.015) | (-0.432 - -0.016) | (-0.385 - 0.189) | (-1.211 - 0.485) |
| Exposure dur.: 7 yrs | -0.356* | -0.320** | -0.273* | not estimable | 0.031 | -0.063 | -0.174 | -0.410 |
|  | (-0.756 - 0.044) | (-0.603 - -0.038) | (-0.586 - 0.041) |  | (-0.243 - 0.305) | (-0.292 - 0.165) | (-0.463 - 0.115) | (-0.952 - 0.133) |
| Exposure dur.: 8 yrs | -0.711*** | -0.394** | -0.524*** | not estimable | -0.137 | -0.303** | -0.413* | -0.520 |
|  | (-1.209 - -0.213) | (-0.731 - -0.057) | (-0.903 - -0.145) |  | (-0.631 - 0.357) | (-0.554 - -0.051) | (-0.879 - 0.054) | (-1.164 - 0.124) |
| Exposure dur.: 9 yrs | 0.134 | -0.266 | -0.329* | not estimable | 0.021 | -0.551 | 0.019 | -0.347 |
|  | (-0.251 - 0.520) | (-0.636 - 0.103) | (-0.700 - 0.042) |  | (-0.353 - 0.396) | (-1.210 - 0.109) | (-0.381 - 0.420) | (-0.859 - 0.165) |
| Exposure dur.: 10 yrs | -0.054 | -0.057 | -0.400* | not estimable | -0.425** | -0.077 | -0.263 | -0.347 |
|  | (-0.664 - 0.556) | (-0.388 - 0.274) | (-0.801 - 0.001) |  | (-0.791 - -0.060) | (-0.566 - 0.412) | (-0.629 - 0.103) | (-0.859 - 0.165) |
| Exposure dur.: 11 yrs | -0.439*** | -0.429 | 0.273 | not estimable | -0.738*** | -1.024*** | -0.112 | -0.361 |
|  | (-0.657 - -0.221) | (-1.054 - 0.196) | (-0.135 - 0.681) |  | (-0.863 - -0.612) | (-1.194 - -0.854) | (-0.558 - 0.335) | (-1.203 - 0.481) |
| Exposure dur.: 12 yrs | - | -0.649*** | -0.931*** | not estimable | - | -2.024*** | 0.311 | -1.361*** |
|  | - | (-1.051 - -0.246) | (-1.213 - -0.649) |  | - | (-2.194 - -1.854) | (-0.571 - 1.194) | (-2.203 - -0.519) |
|  |  |  |  |  |  |  |  |  |
| Observations | 4,674 | 4,141 | 1,954 | 414 | 4,781 | 3,084 | 1,479 | 286 |

SRH - Self-rated Health; German Socio-Economic Panel 1994-2017; Significance levels are *p<0.05, **p<0.01 and ***p<0.001; Corresponds to Figure 1

Shown are the average marginal effects (AME) for the specific exposure duration and the 95% confidence intervals as calculated from panel robust standard errors (SE)

**App. Table 3** Fixed-effects regressions for impact of continuing exposure to housing non-ownership on SRH in different life stages (regression output)

|  | Women | | | | Men | | | |
| --- | --- | --- | --- | --- | --- | --- | --- | --- |
| Impact on Self-rated health | Emerging Adulthood | Early Middle Adulthood | Later Middle Adulthood | Late Adulthood | Emerging Adulthood | Early Middle Adulthood | Later Middle Adulthood | Late Adulthood |
|  |  |  |  |  |  |  |  |  |
| No exposure | Ref. | Ref. | Ref. | Ref. | Ref. | Ref. | Ref. | Ref. |
| Exposure Duration: 1 year | -0.000 | 0.109*** | 0.064 | 0.065 | 0.053*** | 0.184*** | 0.068 | -0.017 |
|  | (0.019) | (0.031) | (0.046) | (0.049) | (0.020) | (0.029) | (0.047) | (0.069) |
| Exposure Duration: 2 years | -0.045** | 0.085*** | 0.065 | 0.006 | -0.005 | 0.152*** | 0.059 | -0.145* |
|  | (0.020) | (0.031) | (0.048) | (0.055) | (0.021) | (0.029) | (0.051) | (0.087) |
| Exposure Duration: 3 years | -0.070*** | 0.029 | 0.041 | -0.035 | -0.040* | 0.091*** | 0.088 | -0.180** |
|  | (0.021) | (0.032) | (0.049) | (0.056) | (0.022) | (0.030) | (0.055) | (0.091) |
| Exposure Duration: 4 years | -0.087*** | 0.006 | -0.011 | -0.077 | -0.079*** | 0.054* | -0.043 | -0.042 |
|  | (0.023) | (0.033) | (0.050) | (0.060) | (0.024) | (0.031) | (0.054) | (0.087) |
| Exposure Duration: 5 years | -0.091*** | -0.034 | -0.002 | -0.143** | -0.061** | 0.003 | -0.065 | -0.136 |
|  | (0.024) | (0.034) | (0.051) | (0.060) | (0.025) | (0.032) | (0.056) | (0.092) |
| Exposure Duration: 6 years | -0.113*** | -0.030 | -0.043 | -0.140** | -0.086*** | 0.010 | -0.084 | -0.129 |
|  | (0.026) | (0.036) | (0.052) | (0.064) | (0.029) | (0.033) | (0.060) | (0.095) |
| Exposure Duration: 7 years | -0.113*** | -0.085** | -0.056 | -0.192*** | -0.101*** | -0.001 | -0.073 | -0.291*** |
|  | (0.030) | (0.037) | (0.053) | (0.067) | (0.030) | (0.035) | (0.063) | (0.107) |
| Exposure Duration: 8 years | -0.102*** | -0.095** | -0.081 | -0.248*** | -0.121*** | -0.051 | -0.131** | -0.161 |
|  | (0.034) | (0.038) | (0.055) | (0.070) | (0.035) | (0.038) | (0.065) | (0.117) |
| Exposure Duration: 9 years | -0.160*** | -0.132*** | -0.074 | -0.303*** | -0.203*** | -0.075* | -0.145** | -0.214* |
|  | (0.037) | (0.041) | (0.060) | (0.075) | (0.040) | (0.039) | (0.070) | (0.122) |
| Exposure Duration: 10 years | -0.127*** | -0.135*** | -0.098 | -0.300*** | -0.184*** | -0.100** | -0.245*** | -0.242* |
|  | (0.041) | (0.044) | (0.063) | (0.074) | (0.045) | (0.042) | (0.073) | (0.127) |
| Exposure Duration: 11 years | -0.156*** | -0.163*** | -0.164** | -0.330*** | -0.246*** | -0.184*** | -0.238*** | -0.386*** |
|  | (0.050) | (0.044) | (0.064) | (0.076) | (0.050) | (0.046) | (0.075) | (0.125) |
| Exposure Duration: 12 years | -0.193*** | -0.199*** | -0.175** | -0.314*** | -0.263*** | -0.194*** | -0.194** | -0.481*** |
|  | (0.060) | (0.047) | (0.068) | (0.077) | (0.059) | (0.048) | (0.079) | (0.136) |
| Exposure Duration: 13 years | -0.287*** | -0.231*** | -0.163** | -0.439*** | -0.246*** | -0.167*** | -0.290*** | -0.548*** |
|  | (0.079) | (0.050) | (0.071) | (0.079) | (0.079) | (0.050) | (0.084) | (0.134) |
| Exposure Duration: 14 years | -0.209* | -0.312*** | -0.201*** | -0.274*** | -0.266*** | -0.272*** | -0.317*** | -0.527*** |
|  | (0.108) | (0.052) | (0.073) | (0.080) | (0.087) | (0.057) | (0.089) | (0.143) |
| Exposure Duration: 15 years | -0.295*** | -0.215*** | -0.163** | -0.490*** | -0.375*** | -0.217*** | -0.323*** | -0.441*** |
|  | (0.100) | (0.054) | (0.078) | (0.091) | (0.121) | (0.060) | (0.096) | (0.139) |
| Full-time employment | Ref. | Ref. | Ref. | Ref. | Ref. | Ref. | Ref. | Ref. |
| Part-time employment | 0.011 | -0.046** | -0.008 | 0.147 | 0.053*** | -0.078** | 0.034 | 0.028 |
|  | (0.017) | (0.021) | (0.031) | (0.134) | (0.019) | (0.039) | (0.050) | (0.091) |
| No employment | 0.060*** | -0.107*** | -0.132*** | -0.004 | -0.008 | -0.185*** | -0.141*** | -0.489*** |
|  | (0.019) | (0.027) | (0.041) | (0.168) | (0.018) | (0.033) | (0.043) | (0.159) |
| Pensioners | -0.031 | -0.282*** | -0.038 | -0.005 | -0.346 | -0.308*** | 0.045 | -0.191** |
|  | (0.262) | (0.086) | (0.041) | (0.138) | (0.281) | (0.110) | (0.048) | (0.083) |
| In education | 0.019 | -0.033 | 0.095 | 0.247 | 0.058*** | -0.083 | -0.115 | 0.131 |
|  | (0.014) | (0.062) | (0.115) | (0.185) | (0.014) | (0.057) | (0.196) | (0.237) |
|  |  |  |  |  |  |  |  |  |
| Low income position | -0.011 | -0.024 | -0.056** | -0.014 | -0.004 | -0.043* | -0.128*** | -0.090* |
|  | (0.013) | (0.019) | (0.025) | (0.030) | (0.014) | (0.024) | (0.034) | (0.049) |
| Medium income position | Ref. | Ref. | Ref. | Ref. | Ref. | Ref. | Ref. | Ref. |
| High income position | 0.018 | -0.010 | 0.083** | 0.026 | 0.002 | -0.015 | 0.017 | 0.043 |
|  | (0.016) | (0.024) | (0.034) | (0.039) | (0.014) | (0.021) | (0.031) | (0.048) |
|  |  |  |  |  |  |  |  |  |
| Single (incl. divorced) | Ref. | Ref. | Ref. | Ref. | Ref. | Ref. | Ref. | Ref. |
| Married | -0.107** | 0.045 | 0.090* | -0.128 | -0.046 | -0.039 | 0.086 | 0.059 |
|  | (0.044) | (0.032) | (0.053) | (0.083) | (0.078) | (0.034) | (0.053) | (0.110) |
| Non-marital partnership | -0.056*** | -0.008 | 0.031 | 0.089 | -0.024** | -0.003 | 0.050 | -0.008 |
|  | (0.012) | (0.019) | (0.029) | (0.055) | (0.012) | (0.022) | (0.036) | (0.052) |
|  |  |  |  |  |  |  |  |  |
| No partner in household | Ref. | Ref. | Ref. | Ref. | Ref. | Ref. | Ref. | Ref. |
| Partner living in household | 0.005 | -0.025 | -0.115** | -0.033 | -0.013 | -0.044 | 0.057 | -0.063 |
|  | (0.017) | (0.025) | (0.057) | (0.104) | (0.019) | (0.027) | (0.042) | (0.092) |
|  |  |  |  |  |  |  |  |  |
| No children<5 in household | Ref. | Ref. | Ref. | Ref. | Ref. | Ref. | Ref. | Ref. |
| Household with children<5 | 0.028 | 0.072*** | 0.121 | -0.119 | 0.014 | 0.005 | 0.015 | 1.014** |
|  | (0.021) | (0.027) | (0.097) | (0.104) | (0.026) | (0.030) | (0.125) | (0.455) |
|  |  |  |  |  |  |  |  |  |
| Western Germany | Ref. | Ref. | Ref. | Ref. | Ref. | Ref. | Ref. | Ref. |
| Eastern Germany | -0.032 | 0.106 | -0.015 | 0.206 | -0.004 | -0.037 | 0.191* | -0.215 |
|  | (0.038) | (0.071) | (0.103) | (0.155) | (0.043) | (0.120) | (0.104) | (0.298) |
|  |  |  |  |  |  |  |  |  |
| Constant | 2.890*** | 2.470*** | 2.162*** | 1.942*** | 2.966*** | 2.599*** | 2.135*** | 2.375*** |
|  | (0.021) | (0.033) | (0.046) | (0.143) | (0.020) | (0.040) | (0.048) | (0.109) |
|  |  |  |  |  |  |  |  |  |
| Observations | 36,265 | 24,642 | 14,463 | 10,400 | 36,279 | 19,587 | 9,849 | 4,610 |

German Socio-Economic Panel 1994-2017; Significance levels are *p<0.05, **p<0.01 and ***p<0.001; Corresponds to Figure 2

Shown are unstandardized beta coefficients (β) and panel robust standard errors (SE)

**App. Table 4** Predictive margins on changes in SRH for each year of continuing exposure towards housing non-ownership in different life stages

|  | Women | | | | | | Men | | | | | | | | |  |  |
| --- | --- | --- | --- | --- | --- | --- | --- | --- | --- | --- | --- | --- | --- | --- | --- | --- | --- |
| Predictive margins | | Emerging Adulthood | Early Middle Adulthood | Later Middle Adulthood | | Late  Adulthood | | | Emerging Adulthood | | Early Middle Adulthood | | Later Middle Adulthood | | Late  Adulthood | | |
| No exposure | | Ref. | Ref. | Ref. | Ref. | | | Ref. | | Ref. | | Ref. | | Ref. | | |  |
| Exposure dur.: 1 yr | | -0.000 | 0.109*** | 0.064 | 0.065 | | | 0.053*** | | 0.184*** | | 0.068 | | -0.017 | | |  |
|  | | (-0.038 - 0.037) | (0.048 - 0.171) | (-0.025 - 0.153) | (-0.031 - 0.162) | | | (0.014 - 0.091) | | (0.127 - 0.240) | | (-0.024 - 0.160) | | (-0.153 - 0.119) | | |  |
| Exposure dur.: 2 yrs | | -0.045** | 0.085*** | 0.065 | 0.006 | | | -0.005 | | 0.152*** | | 0.059 | | -0.145* | | |  |
|  | | (-0.085 - -0.005) | (0.023 - 0.147) | (-0.029 - 0.158) | (-0.101 - 0.113) | | | (-0.045 - 0.036) | | (0.095 - 0.208) | | (-0.041 - 0.158) | | (-0.316 - 0.025) | | |  |
| Exposure dur.: 3 yrs | | -0.070*** | 0.029 | 0.041 | -0.035 | | | -0.040* | | 0.091*** | | 0.088 | | -0.180** | | |  |
|  | | (-0.112 - -0.029) | (-0.034 - 0.091) | (-0.055 - 0.137) | (-0.144 - 0.074) | | | (-0.082 - 0.003) | | (0.033 - 0.149) | | (-0.020 - 0.196) | | (-0.358 - -0.002) | | |  |
| Exposure dur.: 4 yrs | | -0.087*** | 0.006 | -0.011 | -0.077 | | | -0.079*** | | 0.054* | | -0.043 | | -0.042 | | |  |
|  | | (-0.132 - -0.043) | (-0.059 - 0.070) | (-0.109 - 0.087) | (-0.195 - 0.041) | | | (-0.126 - -0.033) | | (-0.007 - 0.115) | | (-0.148 - 0.063) | | (-0.213 - 0.129) | | |  |
| Exposure dur.: 5 yrs | | -0.091*** | -0.034 | -0.002 | -0.143** | | | -0.061** | | 0.003 | | -0.065 | | -0.136 | | |  |
|  | | (-0.139 - -0.044) | (-0.101 - 0.032) | (-0.102 - 0.098) | (-0.260 - -0.026) | | | (-0.111 - -0.012) | | (-0.059 - 0.064) | | (-0.176 - 0.046) | | (-0.317 - 0.045) | | |  |
| Exposure dur.: 6 yrs | | -0.113*** | -0.030 | -0.043 | -0.140** | | | -0.086*** | | 0.010 | | -0.084 | | -0.129 | | |  |
|  | | (-0.165 - -0.061) | (-0.099 - 0.040) | (-0.144 - 0.059) | (-0.266 - -0.014) | | | (-0.142 - -0.029) | | (-0.055 - 0.075) | | (-0.201 - 0.033) | | (-0.315 - 0.056) | | |  |
| Exposure dur.: 7 yrs | | -0.113*** | -0.085** | -0.056 | -0.192*** | | | -0.101*** | | -0.001 | | -0.073 | | -0.291*** | | |  |
|  | | (-0.173 - -0.054) | (-0.157 - -0.013) | (-0.160 - 0.049) | (-0.323 - -0.061) | | | (-0.160 - -0.042) | | (-0.069 - 0.068) | | (-0.197 - 0.050) | | (-0.501 - -0.081) | | |  |
| Exposure dur.: 8 yrs | | -0.102*** | -0.095** | -0.081 | -0.248*** | | | -0.121*** | | -0.051 | | -0.131** | | -0.161 | | |  |
|  | | (-0.168 - -0.035) | (-0.170 - -0.019) | (-0.189 - 0.028) | (-0.386 - -0.111) | | | (-0.189 - -0.053) | | (-0.126 - 0.024) | | (-0.259 - -0.003) | | (-0.390 - 0.068) | | |  |
| Exposure dur.: 9 yrs | | -0.160*** | -0.132*** | -0.074 | -0.303*** | | | -0.203*** | | -0.075* | | -0.145** | | -0.214* | | |  |
|  | | (-0.232 - -0.088) | (-0.211 - -0.052) | (-0.191 - 0.043) | (-0.450 - -0.156) | | | (-0.281 - -0.124) | | (-0.150 - 0.001) | | (-0.282 - -0.009) | | (-0.453 - 0.025) | | |  |
| Exposure dur.: 10 yrs | | -0.127*** | -0.135*** | -0.098 | -0.300*** | | | -0.184*** | | -0.100** | | -0.245*** | | -0.242* | | |  |
|  | | (-0.208 - -0.045) | (-0.221 - -0.049) | (-0.220 - 0.025) | (-0.445 - -0.155) | | | (-0.272 - -0.095) | | (-0.183 - -0.018) | | (-0.389 - -0.102) | | (-0.491 - 0.008) | | |  |
| Exposure dur.: 11 yrs | | -0.156*** | -0.163*** | -0.164** | -0.330*** | | | -0.246*** | | -0.184*** | | -0.238*** | | -0.386*** | | |  |
|  | | (-0.255 - -0.058) | (-0.250 - -0.076) | (-0.288 - -0.039) | (-0.479 - -0.182) | | | (-0.343 - -0.148) | | (-0.274 - -0.093) | | (-0.385 - -0.092) | | (-0.632 - -0.141) | | |  |
| Exposure dur.: 12 yrs | | -0.193*** | -0.199*** | -0.175** | -0.314*** | | | -0.263*** | | -0.194*** | | -0.194** | | -0.481*** | | |  |
|  | | (-0.310 - -0.076) | (-0.291 - -0.107) | (-0.308 - -0.041) | (-0.466 - -0.162) | | | (-0.379 - -0.146) | | (-0.288 - -0.099) | | (-0.349 - -0.039) | | (-0.748 - -0.215) | | |  |
| Exposure dur.: 14 yrs | | -0.287*** | -0.231*** | -0.163** | -0.439*** | | | -0.246*** | | -0.167*** | | -0.290*** | | -0.548*** | | |  |
|  | | (-0.442 - -0.132) | (-0.330 - -0.133) | (-0.302 - -0.023) | (-0.594 - -0.283) | | | (-0.401 - -0.091) | | (-0.265 - -0.068) | | (-0.456 - -0.125) | | (-0.811 - -0.285) | | |  |
| Exposure dur.: 14 yrs | | -0.209* | -0.312*** | -0.201*** | -0.274*** | | | -0.266*** | | -0.272*** | | -0.317*** | | -0.527*** | | |  |
|  | | (-0.420 - 0.003) | (-0.415 - -0.210) | (-0.344 - -0.058) | (-0.431 - -0.116) | | | (-0.436 - -0.096) | | (-0.384 - -0.159) | | (-0.491 - -0.142) | | (-0.807 - -0.246) | | |  |
| Exposure dur.: 15 yrs | | -0.295*** | -0.215*** | -0.163** | -0.490*** | | | -0.375*** | | -0.217*** | | -0.323*** | | -0.441*** | | |  |
|  | | (-0.492 - -0.098) | (-0.320 - -0.110) | (-0.316 - -0.010) | (-0.669 - -0.311) | | | (-0.612 - -0.138) | | (-0.335 - -0.100) | | (-0.511 - -0.135) | | (-0.714 - -0.169) | | |  |
|  | |  |  |  |  | | |  | |  | |  | |  | | |  |
| Observations | | 36,265 | 24,642 | 14,463 | 10,400 | | | 36,279 | | 19,587 | | 9,849 | | 4,610 | | |  |

SRH - Self-rated Health; German Socio-Economic Panel 1994-2017; Significance levels are *p<0.05, **p<0.01 and ***p<0.001; Corresponds to Figure 2

Shown are the average marginal effects (AME) for the specific exposure duration and the 95% confidence intervals as calculated from panel robust standard errors (SE)

**App. Table 5** Fixed-effects regressions for impact of continuing exposure to bad housing quality on SRH in different life stages (regression outputs)

|  | Women | | | | Men | | | |
| --- | --- | --- | --- | --- | --- | --- | --- | --- |
| Impact on Self-rated health | Emerging Adulthood | Early Middle Adulthood | Later Middle Adulthood | Late Adulthood | Emerging Adulthood | Early Middle Adulthood | Later Middle Adulthood | Late Adulthood |
|  |  |  |  |  |  |  |  |  |
| No exposure | Ref. | Ref. | Ref. | Ref. | Ref. | Ref. | Ref. | Ref. |
| Exposure Duration: 1 year | -0.030** | 0.023 | -0.057** | 0.023 | 0.009 | 0.010 | 0.007 | -0.020 |
|  | (0.013) | (0.017) | (0.022) | (0.027) | (0.013) | (0.018) | (0.028) | (0.039) |
| Exposure Duration: 2 years | -0.061*** | -0.015 | -0.050* | 0.017 | -0.036** | -0.039 | -0.069** | -0.067 |
|  | (0.017) | (0.021) | (0.030) | (0.035) | (0.017) | (0.024) | (0.035) | (0.060) |
| Exposure Duration: 3 years | -0.045** | -0.033 | -0.040 | -0.028 | -0.067*** | -0.044 | 0.018 | -0.010 |
|  | (0.021) | (0.025) | (0.035) | (0.040) | (0.021) | (0.029) | (0.045) | (0.068) |
| Exposure Duration: 4 years | -0.085*** | -0.082*** | -0.091** | -0.021 | -0.060** | -0.078** | -0.045 | -0.089 |
|  | (0.026) | (0.031) | (0.041) | (0.045) | (0.025) | (0.034) | (0.048) | (0.085) |
| Exposure Duration: 5 years | -0.102*** | -0.049 | -0.158*** | -0.037 | -0.040 | -0.098** | -0.038 | -0.130 |
|  | (0.032) | (0.033) | (0.046) | (0.054) | (0.032) | (0.040) | (0.058) | (0.091) |
| Exposure Duration: 6 years | -0.147*** | -0.055 | -0.157*** | 0.018 | -0.056 | -0.084* | -0.063 | -0.191* |
|  | (0.042) | (0.040) | (0.059) | (0.062) | (0.036) | (0.045) | (0.063) | (0.109) |
| Exposure Duration: 7 years | -0.104* | -0.156*** | -0.105 | -0.045 | -0.112** | -0.118** | -0.055 | 0.028 |
|  | (0.055) | (0.048) | (0.066) | (0.074) | (0.047) | (0.048) | (0.072) | (0.116) |
| Exposure Duration: 8 years | -0.056 | -0.232*** | -0.245*** | -0.080 | -0.167*** | -0.149** | -0.133* | -0.074 |
|  | (0.066) | (0.057) | (0.070) | (0.080) | (0.061) | (0.060) | (0.069) | (0.124) |
| Exposure Duration: 9 years | -0.119 | -0.115* | -0.167** | -0.177* | -0.062 | -0.119* | -0.166* | -0.043 |
|  | (0.100) | (0.062) | (0.083) | (0.100) | (0.071) | (0.064) | (0.096) | (0.110) |
| Exposure Duration: 10 years | -0.031 | -0.218*** | -0.115 | -0.202* | -0.178* | -0.228*** | -0.272** | -0.030 |
|  | (0.088) | (0.074) | (0.085) | (0.110) | (0.093) | (0.080) | (0.108) | (0.131) |
| Exposure Duration: 11 years | -0.046 | -0.257*** | -0.264*** | 0.095 | -0.330** | -0.214** | -0.252** | -0.301** |
|  | (0.165) | (0.090) | (0.093) | (0.111) | (0.134) | (0.092) | (0.113) | (0.145) |
| Exposure Duration: 12 years | -0.167 | -0.173 | -0.285*** | 0.073 | -0.379*** | -0.315*** | -0.424*** | -0.352** |
|  | (0.161) | (0.110) | (0.096) | (0.133) | (0.131) | (0.118) | (0.116) | (0.148) |
| Exposure Duration: 13 years | -0.289 | -0.396*** | -0.280*** | -0.000 | -0.067 | -0.438*** | -0.479*** | -0.271 |
|  | (0.264) | (0.124) | (0.093) | (0.144) | (0.242) | (0.124) | (0.161) | (0.175) |
| Exposure Duration: 14 years | 0.247 | -0.276** | -0.246** | -0.258 | -0.220 | -0.281** | -0.624*** | -0.458** |
|  | (0.244) | (0.139) | (0.097) | (0.188) | (0.361) | (0.115) | (0.176) | (0.207) |
| Exposure Duration: 15 years | -0.167*** | -0.227 | -0.183* | -0.063 | -0.720*** | -0.244* | -0.602*** | -0.313* |
|  | (0.058) | (0.167) | (0.102) | (0.210) | (0.040) | (0.125) | (0.187) | (0.174) |

| Full-time employment | Ref. | Ref. | Ref. | Ref. | Ref. | Ref. | Ref. | Ref. |
| --- | --- | --- | --- | --- | --- | --- | --- | --- |
| Part-time employment | 0.021 | -0.038* | -0.030 | 0.188 | 0.068*** | -0.092** | -0.036 | 0.074 |
|  | (0.018) | (0.022) | (0.033) | (0.143) | (0.020) | (0.042) | (0.052) | (0.096) |
| No employment | 0.067*** | -0.076*** | -0.165*** | 0.013 | -0.001 | -0.212*** | -0.172*** | -0.386** |
|  | (0.020) | (0.027) | (0.041) | (0.175) | (0.019) | (0.035) | (0.045) | (0.172) |
| Pensioners | 0.007 | -0.331*** | -0.097** | -0.001 | -0.232 | -0.363*** | -0.017 | -0.177** |
|  | (0.270) | (0.089) | (0.042) | (0.144) | (0.261) | (0.106) | (0.052) | (0.084) |
| In education | 0.046*** | -0.018 | 0.075 | 0.198 | 0.087*** | -0.066 | -0.194 | 0.166 |
|  | (0.015) | (0.070) | (0.134) | (0.197) | (0.014) | (0.059) | (0.195) | (0.249) |
|  |  |  |  |  |  |  |  |  |
| Low income position | -0.011 | -0.017 | -0.059** | -0.016 | 0.006 | -0.019 | -0.133*** | -0.116** |
|  | (0.014) | (0.019) | (0.026) | (0.030) | (0.014) | (0.026) | (0.036) | (0.049) |
| Medium income position | Ref. | Ref. | Ref. | Ref. | Ref. | Ref. | Ref. | Ref. |
| High income position | 0.029* | -0.024 | 0.076** | 0.014 | -0.005 | -0.019 | 0.018 | -0.016 |
|  | (0.016) | (0.025) | (0.035) | (0.043) | (0.015) | (0.022) | (0.033) | (0.049) |
|  |  |  |  |  |  |  |  |  |
| Single (incl. divorced) | Ref. | Ref. | Ref. | Ref. | Ref. | Ref. | Ref. | Ref. |
| Married | -0.107** | 0.097*** | 0.173*** | -0.004 | -0.008 | 0.004 | 0.082 | 0.169 |
|  | (0.046) | (0.032) | (0.055) | (0.088) | (0.079) | (0.035) | (0.054) | (0.131) |
| Non-marital partnership | -0.060*** | -0.018 | 0.042 | 0.081 | -0.026** | -0.003 | 0.049 | 0.008 |
|  | (0.013) | (0.020) | (0.030) | (0.055) | (0.012) | (0.023) | (0.038) | (0.055) |
|  |  |  |  |  |  |  |  |  |
| No partner in household | Ref. | Ref. | Ref. | Ref. | Ref. | Ref. | Ref. | Ref. |
| Partner living in household | -0.006 | 0.006 | -0.105* | 0.093 | -0.028 | -0.052* | 0.028 | -0.075 |
|  | (0.017) | (0.026) | (0.057) | (0.104) | (0.019) | (0.028) | (0.043) | (0.096) |
|  |  |  |  |  |  |  |  |  |
| No children<5 in household | Ref. | Ref. | Ref. | Ref. | Ref. | Ref. | Ref. | Ref. |
| Children<5 in household = 1, Household with children<5 | 0.014 | 0.080*** | 0.144 | -0.111 | 0.011 | 0.036 | 0.006 | 1.017** |
|  | (0.023) | (0.028) | (0.093) | (0.094) | (0.027) | (0.033) | (0.117) | (0.451) |
|  |  |  |  |  |  |  |  |  |
| Western Germany | Ref. | Ref. | Ref. | Ref. | Ref. | Ref. | Ref. | Ref. |
| Eastern Germany | -0.030 | 0.140* | -0.007 | 0.224 | 0.011 | -0.050 | 0.235** | -0.172 |
|  | (0.040) | (0.074) | (0.105) | (0.157) | (0.044) | (0.121) | (0.102) | (0.328) |
|  |  |  |  |  |  |  |  |  |
| Constant | 2.863*** | 2.445*** | 2.184*** | 1.825*** | 2.942*** | 2.627*** | 2.123*** | 2.273*** |
|  | (0.017) | (0.027) | (0.037) | (0.149) | (0.017) | (0.039) | (0.040) | (0.122) |
|  |  |  |  |  |  |  |  |  |
| Observations | 33,018 | 22,825 | 13,295 | 10,142 | 33,082 | 18,655 | 9,215 | 4,436 |

German Socio-Economic Panel 1994-2017; Significance levels are *p<0.05, **p<0.01 and ***p<0.001; Corresponds to Figure 3

Shown are unstandardized beta coefficients (β) and panel robust standard errors (SE)

**App. Table 6** Predictive margins on changes in SRH for each year of continuing exposure towards bad housing quality in different life stages

|  | Women | | | | Men | | | |
| --- | --- | --- | --- | --- | --- | --- | --- | --- |
| Predictive margins | Emerging Adulthood | Early Middle Adulthood | Later Middle Adulthood | Late  Adulthood | Emerging Adulthood | Early Middle Adulthood | Later Middle Adulthood | Late  Adulthood |
| No exposure | Ref. | Ref. | Ref. | Ref. | Ref. | Ref. | Ref. | Ref. |
| Exposure dur.: 1 yr | -0.030** | 0.023 | -0.057** | 0.023 | 0.009 | 0.010 | 0.007 | -0.020 |
|  | (-0.055 - -0.005) | (-0.011 - 0.056) | (-0.101 - -0.013) | (-0.029 - 0.075) | (-0.015 - 0.034) | (-0.025 - 0.044) | (-0.047 - 0.061) | (-0.097 - 0.056) |
| Exposure dur.: 2 yrs | -0.061*** | -0.015 | -0.050* | 0.017 | -0.036** | -0.039 | -0.069** | -0.067 |
|  | (-0.093 - -0.028) | (-0.057 - 0.026) | (-0.109 - 0.009) | (-0.051 - 0.085) | (-0.069 - -0.003) | (-0.086 - 0.008) | (-0.137 - -0.001) | (-0.184 - 0.050) |
| Exposure dur.: 3 yrs | -0.045** | -0.033 | -0.040 | -0.028 | -0.067*** | -0.044 | 0.018 | -0.010 |
|  | (-0.086 - -0.003) | (-0.083 - 0.017) | (-0.107 - 0.028) | (-0.106 - 0.050) | (-0.108 - -0.025) | (-0.101 - 0.013) | (-0.071 - 0.106) | (-0.144 - 0.124) |
| Exposure dur.: 4 yrs | -0.085*** | -0.082*** | -0.091** | -0.021 | -0.060** | -0.078** | -0.045 | -0.089 |
|  | (-0.136 - -0.034) | (-0.143 - -0.022) | (-0.171 - -0.012) | (-0.109 - 0.068) | (-0.110 - -0.011) | (-0.144 - -0.011) | (-0.139 - 0.049) | (-0.256 - 0.078) |
| Exposure dur.: 5 yrs | -0.102*** | -0.049 | -0.158*** | -0.037 | -0.040 | -0.098** | -0.038 | -0.130 |
|  | (-0.165 - -0.038) | (-0.115 - 0.016) | (-0.248 - -0.068) | (-0.143 - 0.068) | (-0.102 - 0.023) | (-0.177 - -0.019) | (-0.152 - 0.076) | (-0.309 - 0.049) |
| Exposure dur.: 6 yrs | -0.147*** | -0.055 | -0.157*** | 0.018 | -0.056 | -0.084* | -0.063 | -0.191* |
|  | (-0.229 - -0.066) | (-0.134 - 0.024) | (-0.274 - -0.041) | (-0.104 - 0.139) | (-0.126 - 0.015) | (-0.171 - 0.003) | (-0.186 - 0.060) | (-0.405 - 0.023) |
| Exposure dur.: 7 yrs | -0.104* | -0.156*** | -0.105 | -0.045 | -0.112** | -0.118** | -0.055 | 0.028 |
|  | (-0.210 - 0.003) | (-0.250 - -0.063) | (-0.234 - 0.023) | (-0.191 - 0.101) | (-0.205 - -0.019) | (-0.213 - -0.023) | (-0.195 - 0.085) | (-0.199 - 0.256) |
| Exposure dur.: 8 yrs | -0.056 | -0.232*** | -0.245*** | -0.080 | -0.167*** | -0.149** | -0.133* | -0.074 |
|  | (-0.186 - 0.073) | (-0.342 - -0.121) | (-0.382 - -0.108) | (-0.236 - 0.077) | (-0.286 - -0.047) | (-0.266 - -0.032) | (-0.268 - 0.001) | (-0.317 - 0.169) |
| Exposure dur.: 9 yrs | -0.119 | -0.115* | -0.167** | -0.177* | -0.062 | -0.119* | -0.166* | -0.043 |
|  | (-0.316 - 0.078) | (-0.237 - 0.006) | (-0.329 - -0.006) | (-0.373 - 0.019) | (-0.201 - 0.077) | (-0.246 - 0.007) | (-0.354 - 0.023) | (-0.258 - 0.172) |
| Exposure dur.: 10 yrs | -0.031 | -0.218*** | -0.115 | -0.202* | -0.178* | -0.228*** | -0.272** | -0.030 |
|  | (-0.204 - 0.142) | (-0.364 - -0.072) | (-0.282 - 0.053) | (-0.417 - 0.013) | (-0.360 - 0.004) | (-0.386 - -0.070) | (-0.485 - -0.060) | (-0.287 - 0.227) |
| Exposure dur.: 11 yrs | -0.046 | -0.257*** | -0.264*** | 0.095 | -0.330** | -0.214** | -0.252** | -0.301** |
|  | (-0.369 - 0.278) | (-0.432 - -0.081) | (-0.446 - -0.083) | (-0.122 - 0.313) | (-0.593 - -0.068) | (-0.394 - -0.034) | (-0.474 - -0.031) | (-0.585 - -0.016) |
| Exposure dur.: 12 yrs | -0.167 | -0.173 | -0.285*** | 0.073 | -0.379*** | -0.315*** | -0.424*** | -0.352** |
|  | (-0.483 - 0.148) | (-0.388 - 0.042) | (-0.473 - -0.097) | (-0.188 - 0.335) | (-0.636 - -0.121) | (-0.546 - -0.084) | (-0.651 - -0.196) | (-0.642 - -0.061) |
| Exposure dur.: 14 yrs | -0.289 | -0.396*** | -0.280*** | -0.000 | -0.067 | -0.438*** | -0.479*** | -0.271 |
|  | (-0.807 - 0.229) | (-0.638 - -0.154) | (-0.463 - -0.097) | (-0.282 - 0.282) | (-0.540 - 0.407) | (-0.681 - -0.196) | (-0.795 - -0.163) | (-0.615 - 0.073) |
| Exposure dur.: 14 yrs | 0.247 | -0.276** | -0.246** | -0.258 | -0.220 | -0.281** | -0.624*** | -0.458** |
|  | (-0.230 - 0.725) | (-0.549 - -0.004) | (-0.435 - -0.057) | (-0.627 - 0.110) | (-0.928 - 0.487) | (-0.506 - -0.056) | (-0.968 - -0.279) | (-0.865 - -0.052) |
| Exposure dur.: 15 yrs | -0.167*** | -0.227 | -0.183* | -0.063 | -0.720*** | -0.244* | -0.602*** | -0.313* |
|  | (-0.280 - -0.054) | (-0.555 - 0.100) | (-0.383 - 0.018) | (-0.475 - 0.348) | (-0.800 - -0.641) | (-0.488 - 0.001) | (-0.969 - -0.235) | (-0.654 - 0.028) |
| Observations | 33,018 | 22,825 | 13,295 | 10,142 | 33,082 | 18,655 | 9,215 | 4,436 |

SRH - Self-rated Health; German Socio-Economic Panel 1994-2017; Significance levels are *p<0.05, **p<0.01 and ***p<0.001; Corresponds to Figure 3

Shown are the average marginal effects (AME) for the specific exposure duration and the 95% confidence intervals as calculated from panel robust standard errors (SE)

**App. Table 7** Fixed-effects regressions for impact of continuing exposure to financial worries on SRH in different life stages (regression outputs)

|  | Women | | | | Men | | | |
| --- | --- | --- | --- | --- | --- | --- | --- | --- |
| Impact on Self-rated health | Emerging Adulthood | Early Middle Adulthood | Later Middle Adulthood | Late Adulthood | Emerging Adulthood | Early Middle Adulthood | Later Middle Adulthood | Late Adulthood |
|  |  |  |  |  |  |  |  |  |
| No exposure | Ref. | Ref. | Ref. | Ref. | Ref. | Ref. | Ref. | Ref. |
| Exposure Duration: 1 year | -0.078*** | -0.034* | -0.072*** | 0.015 | -0.044*** | -0.040** | -0.090*** | -0.054* |
|  | (0.013) | (0.019) | (0.021) | (0.021) | (0.012) | (0.017) | (0.025) | (0.031) |
| Exposure Duration: 2 years | -0.133*** | -0.065*** | -0.081*** | 0.006 | -0.102*** | -0.071*** | -0.096*** | -0.040 |
|  | (0.015) | (0.022) | (0.026) | (0.027) | (0.014) | (0.020) | (0.032) | (0.042) |
| Exposure Duration: 3 years | -0.153*** | -0.093*** | -0.098*** | 0.010 | -0.127*** | -0.114*** | -0.110*** | -0.114** |
|  | (0.017) | (0.024) | (0.028) | (0.033) | (0.016) | (0.022) | (0.035) | (0.051) |
| Exposure Duration: 4 years | -0.148*** | -0.117*** | -0.126*** | 0.009 | -0.180*** | -0.145*** | -0.173*** | -0.038 |
|  | (0.019) | (0.026) | (0.031) | (0.036) | (0.018) | (0.025) | (0.040) | (0.058) |
| Exposure Duration: 5 years | -0.148*** | -0.142*** | -0.104*** | -0.036 | -0.148*** | -0.192*** | -0.158*** | -0.004 |
|  | (0.021) | (0.028) | (0.033) | (0.038) | (0.020) | (0.028) | (0.041) | (0.056) |
| Exposure Duration: 6 years | -0.158*** | -0.171*** | -0.138*** | -0.089** | -0.189*** | -0.172*** | -0.137*** | -0.055 |
|  | (0.023) | (0.030) | (0.037) | (0.043) | (0.024) | (0.030) | (0.045) | (0.075) |
| Exposure Duration: 7 years | -0.145*** | -0.204*** | -0.131*** | -0.125** | -0.229*** | -0.209*** | -0.180*** | -0.093 |
|  | (0.026) | (0.032) | (0.039) | (0.050) | (0.028) | (0.033) | (0.049) | (0.073) |
| Exposure Duration: 8 years | -0.155*** | -0.219*** | -0.162*** | -0.172*** | -0.245*** | -0.241*** | -0.208*** | -0.112 |
|  | (0.030) | (0.034) | (0.041) | (0.051) | (0.030) | (0.037) | (0.051) | (0.084) |
| Exposure Duration: 9 years | -0.196*** | -0.264*** | -0.212*** | -0.170*** | -0.287*** | -0.298*** | -0.200*** | -0.051 |
|  | (0.034) | (0.039) | (0.045) | (0.056) | (0.037) | (0.039) | (0.059) | (0.087) |
| Exposure Duration: 10 years | -0.199*** | -0.204*** | -0.149*** | -0.131** | -0.303*** | -0.306*** | -0.238*** | -0.179 |
|  | (0.042) | (0.042) | (0.049) | (0.056) | (0.042) | (0.041) | (0.063) | (0.109) |
| Exposure Duration: 11 years | -0.174*** | -0.234*** | -0.210*** | -0.255*** | -0.251*** | -0.359*** | -0.269*** | -0.349*** |
|  | (0.048) | (0.045) | (0.049) | (0.073) | (0.049) | (0.047) | (0.060) | (0.107) |
| Exposure Duration: 12 years | -0.220*** | -0.298*** | -0.280*** | -0.155** | -0.387*** | -0.397*** | -0.265*** | -0.299** |
|  | (0.055) | (0.048) | (0.053) | (0.067) | (0.061) | (0.052) | (0.069) | (0.118) |
| Exposure Duration: 13 years | -0.327*** | -0.300*** | -0.139*** | -0.171** | -0.270*** | -0.421*** | -0.298*** | -0.206* |
|  | (0.071) | (0.050) | (0.053) | (0.069) | (0.074) | (0.052) | (0.073) | (0.117) |
| Exposure Duration: 14 years | -0.399*** | -0.333*** | -0.264*** | -0.076 | -0.239** | -0.474*** | -0.338*** | -0.189 |
|  | (0.090) | (0.054) | (0.059) | (0.082) | (0.101) | (0.059) | (0.085) | (0.132) |
| Exposure Duration: 15 years | -0.315*** | -0.258*** | -0.146** | -0.108 | -0.304** | -0.369*** | -0.321*** | -0.177 |
|  | (0.089) | (0.057) | (0.061) | (0.084) | (0.133) | (0.062) | (0.092) | (0.135) |
| Full-time employment | Ref. | Ref. | Ref. | Ref. | Ref. | Ref. | Ref. | Ref. |
| Part-time employment | 0.026 | -0.034 | -0.008 | 0.146 | 0.063*** | -0.073* | -0.018 | 0.028 |
|  | (0.017) | (0.021) | (0.031) | (0.143) | (0.019) | (0.039) | (0.049) | (0.089) |
| No employment | 0.075*** | -0.095*** | -0.145*** | 0.008 | -0.001 | -0.184*** | -0.157*** | -0.388** |
|  | (0.019) | (0.027) | (0.041) | (0.175) | (0.018) | (0.033) | (0.043) | (0.170) |
| Pensioners | -0.043 | -0.283*** | -0.085** | -0.041 | -0.348 | -0.286*** | -0.018 | -0.210** |
|  | (0.269) | (0.083) | (0.041) | (0.145) | (0.291) | (0.109) | (0.051) | (0.082) |
| In education | 0.036** | -0.008 | 0.082 | 0.234 | 0.060*** | -0.067 | -0.174 | 0.152 |
|  | (0.014) | (0.062) | (0.114) | (0.191) | (0.014) | (0.057) | (0.176) | (0.240) |
|  |  |  |  |  |  |  |  |  |
| Low income position | -0.007 | -0.007 | -0.047* | -0.008 | 0.009 | -0.017 | -0.107*** | -0.073 |
|  | (0.013) | (0.019) | (0.025) | (0.029) | (0.014) | (0.024) | (0.036) | (0.048) |
| Medium income position | Ref. | Ref. | Ref. | Ref. | Ref. | Ref. | Ref. | Ref. |
| High income position | 0.019 | -0.036 | 0.052 | 0.027 | -0.005 | -0.034 | 0.011 | 0.004 |
|  | (0.016) | (0.025) | (0.034) | (0.038) | (0.014) | (0.021) | (0.032) | (0.049) |
|  |  |  |  |  |  |  |  |  |
| Single (incl. divorced) | Ref. | Ref. | Ref. | Ref. | Ref. | Ref. | Ref. | Ref. |
| Family status = 1, Married | -0.115*** | 0.055* | 0.112** | -0.043 | -0.050 | -0.011 | 0.098* | 0.008 |
|  | (0.044) | (0.031) | (0.054) | (0.085) | (0.078) | (0.033) | (0.053) | (0.115) |
| Non-marital partnership | -0.053*** | -0.014 | 0.033 | 0.086 | -0.021* | -0.006 | 0.070** | -0.016 |
|  | (0.012) | (0.019) | (0.028) | (0.054) | (0.012) | (0.022) | (0.035) | (0.051) |
|  |  |  |  |  |  |  |  |  |
| No partner in household | Ref. | Ref. | Ref. | Ref. | Ref. | Ref. | Ref. | Ref. |
| Partner in household = 1, Partner living in household | -0.004 | -0.020 | -0.090* | 0.016 | -0.017 | -0.036 | 0.027 | 0.001 |
|  | (0.017) | (0.025) | (0.052) | (0.099) | (0.018) | (0.027) | (0.042) | (0.081) |
|  |  |  |  |  |  |  |  |  |
| No children<5 in household | Ref. | Ref. | Ref. | Ref. | Ref. | Ref. | Ref. | Ref. |
| Household with children<5 | 0.029 | 0.074*** | 0.093 | -0.063 | 0.016 | 0.016 | 0.012 | 1.005** |
|  | (0.021) | (0.027) | (0.097) | (0.093) | (0.026) | (0.031) | (0.126) | (0.439) |
|  |  |  |  |  |  |  |  |  |
| Western Germany | Ref. | Ref. | Ref. | Ref. | Ref. | Ref. | Ref. | Ref. |
| Eastern Germany | -0.019 | 0.109 | 0.019 | 0.206 | -0.001 | -0.074 | 0.158* | -0.501 |
|  | (0.038) | (0.069) | (0.102) | (0.145) | (0.043) | (0.104) | (0.090) | (0.349) |
|  |  |  |  |  |  |  |  |  |
| Constant | 2.932*** | 2.549*** | 2.238*** | 1.912*** | 3.025*** | 2.739*** | 2.230*** | 2.412*** |
|  | (0.019) | (0.029) | (0.039) | (0.149) | (0.018) | (0.037) | (0.039) | (0.120) |
|  |  |  |  |  |  |  |  |  |
| Observations | 36,101 | 24,764 | 14,654 | 10,687 | 36,115 | 19,820 | 9,978 | 4,840 |

German Socio-Economic Panel 1994-2017; Significance levels are *p<0.05, **p<0.01 and ***p<0.001; Corresponds to Figure 4a

Shown are unstandardized beta coefficients (β) and panel robust standard errors (SE)

**App. Table 8** Predictive margins on changes in SRH for each year of continuing exposure towards financial worries in different life stages

|  | Women | | | | | Men | | | | |  |
| --- | --- | --- | --- | --- | --- | --- | --- | --- | --- | --- | --- |
| Predictive margins | | Emerging Adulthood | Early Middle Adulthood | Later Middle Adulthood | Late  Adulthood | | Emerging Adulthood | Early Middle Adulthood | Later Middle Adulthood | Late  Adulthood | |
| No exposure | | Ref. | Ref. | Ref. | Ref. | | Ref. | Ref. | Ref. | Ref. | |
| Exposure dur.: 1 yr | | -0.078*** | -0.034* | -0.072*** | 0.015 | | -0.044*** | -0.040** | -0.090*** | -0.054* | |
|  | | (-0.103 - -0.053) | (-0.071 - 0.002) | (-0.113 - -0.030) | (-0.026 - 0.057) | | (-0.067 - -0.021) | (-0.073 - -0.007) | (-0.139 - -0.041) | (-0.114 - 0.006) | |
| Exposure dur.: 2 yrs | | -0.133*** | -0.065*** | -0.081*** | 0.006 | | -0.102*** | -0.071*** | -0.096*** | -0.040 | |
|  | | (-0.162 - -0.105) | (-0.107 - -0.022) | (-0.132 - -0.030) | (-0.047 - 0.059) | | (-0.129 - -0.075) | (-0.110 - -0.031) | (-0.158 - -0.034) | (-0.123 - 0.043) | |
| Exposure dur.: 3 yrs | | -0.153*** | -0.093*** | -0.098*** | 0.010 | | -0.127*** | -0.114*** | -0.110*** | -0.114** | |
|  | | (-0.186 - -0.121) | (-0.140 - -0.047) | (-0.153 - -0.043) | (-0.055 - 0.074) | | (-0.158 - -0.096) | (-0.158 - -0.070) | (-0.179 - -0.042) | (-0.213 - -0.015) | |
| Exposure dur.: 4 yrs | | -0.148*** | -0.117*** | -0.126*** | 0.009 | | -0.180*** | -0.145*** | -0.173*** | -0.038 | |
|  | | (-0.184 - -0.111) | (-0.167 - -0.067) | (-0.187 - -0.066) | (-0.061 - 0.079) | | (-0.215 - -0.144) | (-0.194 - -0.096) | (-0.251 - -0.095) | (-0.152 - 0.075) | |
| Exposure dur.: 5 yrs | | -0.148*** | -0.142*** | -0.104*** | -0.036 | | -0.148*** | -0.192*** | -0.158*** | -0.004 | |
|  | | (-0.189 - -0.106) | (-0.196 - -0.087) | (-0.169 - -0.039) | (-0.111 - 0.039) | | (-0.187 - -0.108) | (-0.246 - -0.138) | (-0.237 - -0.078) | (-0.114 - 0.106) | |
| Exposure dur.: 6 yrs | | -0.158*** | -0.171*** | -0.138*** | -0.089** | | -0.189*** | -0.172*** | -0.137*** | -0.055 | |
|  | | (-0.204 - -0.112) | (-0.230 - -0.112) | (-0.211 - -0.066) | (-0.173 - -0.004) | | (-0.237 - -0.141) | (-0.231 - -0.112) | (-0.224 - -0.049) | (-0.202 - 0.092) | |
| Exposure dur.: 7 yrs | | -0.145*** | -0.204*** | -0.131*** | -0.125** | | -0.229*** | -0.209*** | -0.180*** | -0.093 | |
|  | | (-0.196 - -0.094) | (-0.268 - -0.141) | (-0.207 - -0.054) | (-0.224 - -0.026) | | (-0.283 - -0.174) | (-0.274 - -0.144) | (-0.276 - -0.084) | (-0.237 - 0.051) | |
| Exposure dur.: 8 yrs | | -0.155*** | -0.219*** | -0.162*** | -0.172*** | | -0.245*** | -0.241*** | -0.208*** | -0.112 | |
|  | | (-0.215 - -0.095) | (-0.286 - -0.152) | (-0.242 - -0.082) | (-0.272 - -0.071) | | (-0.305 - -0.186) | (-0.314 - -0.168) | (-0.307 - -0.108) | (-0.276 - 0.052) | |
| Exposure dur.: 9 yrs | | -0.196*** | -0.264*** | -0.212*** | -0.170*** | | -0.287*** | -0.298*** | -0.200*** | -0.051 | |
|  | | (-0.263 - -0.128) | (-0.340 - -0.188) | (-0.299 - -0.124) | (-0.280 - -0.060) | | (-0.360 - -0.215) | (-0.374 - -0.222) | (-0.315 - -0.085) | (-0.220 - 0.119) | |
| Exposure dur.: 10 yrs | | -0.199*** | -0.204*** | -0.149*** | -0.131** | | -0.303*** | -0.306*** | -0.238*** | -0.179 | |
|  | | (-0.280 - -0.117) | (-0.286 - -0.121) | (-0.245 - -0.053) | (-0.241 - -0.022) | | (-0.385 - -0.221) | (-0.387 - -0.225) | (-0.361 - -0.115) | (-0.394 - 0.035) | |
| Exposure dur.: 11 yrs | | -0.174*** | -0.234*** | -0.210*** | -0.255*** | | -0.251*** | -0.359*** | -0.269*** | -0.349*** | |
|  | | (-0.268 - -0.080) | (-0.322 - -0.145) | (-0.307 - -0.114) | (-0.398 - -0.111) | | (-0.346 - -0.155) | (-0.451 - -0.267) | (-0.387 - -0.150) | (-0.557 - -0.140) | |
| Exposure dur.: 12 yrs | | -0.220*** | -0.298*** | -0.280*** | -0.155** | | -0.387*** | -0.397*** | -0.265*** | -0.299** | |
|  | | (-0.327 - -0.113) | (-0.391 - -0.204) | (-0.384 - -0.177) | (-0.286 - -0.024) | | (-0.506 - -0.268) | (-0.499 - -0.295) | (-0.400 - -0.130) | (-0.531 - -0.068) | |
| Exposure dur.: 14 yrs | | -0.327*** | -0.300*** | -0.139*** | -0.171** | | -0.270*** | -0.421*** | -0.298*** | -0.206* | |
|  | | (-0.467 - -0.188) | (-0.398 - -0.203) | (-0.243 - -0.035) | (-0.306 - -0.036) | | (-0.415 - -0.125) | (-0.522 - -0.320) | (-0.441 - -0.155) | (-0.436 - 0.025) | |
| Exposure dur.: 14 yrs | | -0.399*** | -0.333*** | -0.264*** | -0.076 | | -0.239** | -0.474*** | -0.338*** | -0.189 | |
|  | | (-0.574 - -0.223) | (-0.439 - -0.227) | (-0.379 - -0.148) | (-0.236 - 0.084) | | (-0.437 - -0.041) | (-0.590 - -0.358) | (-0.503 - -0.172) | (-0.447 - 0.069) | |
| Exposure dur.: 15 yrs | | -0.315*** | -0.258*** | -0.146** | -0.108 | | -0.304** | -0.369*** | -0.321*** | -0.177 | |
|  | | (-0.490 - -0.141) | (-0.369 - -0.146) | (-0.266 - -0.025) | (-0.273 - 0.058) | | (-0.565 - -0.043) | (-0.491 - -0.247) | (-0.501 - -0.141) | (-0.442 - 0.088) | |
|  | |  |  |  |  | |  |  |  |  | |
| Observations | | 36,101 | 24,764 | 14,654 | 10,687 | | 36,115 | 19,820 | 9,978 | 4,840 | |

SRH - Self-rated Health; German Socio-Economic Panel 1994-2017; Significance levels are *p<0.05, **p<0.01 and ***p<0.001; Corresponds to Figure 4a

Shown are the average marginal effects (AME) for the specific exposure duration and the 95% confidence intervals as calculated from panel robust standard errors (SE)

**App. Table 9** Fixed-effects regressions for impact of continuing exposure to occupational worries on SRH in different life stages (regression outputs)

|  | Women | | | | Men | | | |
| --- | --- | --- | --- | --- | --- | --- | --- | --- |
| Impact on Self-rated health | Emerging Adulthood | Early Middle Adulthood | Later Middle Adulthood | Late Adulthood | Emerging Adulthood | Early Middle Adulthood | Later Middle Adulthood | Late Adulthood |
|  |  |  |  |  |  |  |  |  |
| No exposure | Ref. | Ref. | Ref. | Ref. | Ref. | Ref. | Ref. | Ref. |
| Exposure Duration: 1 year | -0.046*** | -0.050*** | 0.009 | 0.044 | -0.025* | -0.053*** | -0.044 | 0.170 |
|  | (0.013) | (0.017) | (0.024) | (0.100) | (0.013) | (0.017) | (0.027) | (0.130) |
| Exposure Duration: 2 years | -0.060*** | -0.059*** | 0.020 | 0.212 | -0.077*** | -0.055*** | -0.075** | -0.316 |
|  | (0.018) | (0.021) | (0.032) | (0.173) | (0.017) | (0.021) | (0.034) | (0.235) |
| Exposure Duration: 3 years | -0.054** | -0.093*** | -0.025 | 0.388** | -0.136*** | -0.080*** | -0.052 | -0.331 |
|  | (0.022) | (0.025) | (0.040) | (0.170) | (0.022) | (0.026) | (0.043) | (0.206) |
| Exposure Duration: 4 years | -0.075*** | -0.118*** | -0.081* | 0.680** | -0.136*** | -0.082*** | -0.066 | -0.055 |
|  | (0.028) | (0.028) | (0.046) | (0.295) | (0.028) | (0.030) | (0.050) | (0.184) |
| Exposure Duration: 5 years | -0.067* | -0.138*** | -0.072 | 0.864 | -0.104*** | -0.093*** | -0.005 | -1.147*** |
|  | (0.036) | (0.033) | (0.048) | (0.654) | (0.034) | (0.035) | (0.053) | (0.197) |
| Exposure Duration: 6 years | -0.093** | -0.178*** | -0.054 | 0.436 | -0.203*** | -0.115*** | -0.003 | - |
|  | (0.041) | (0.041) | (0.057) | (0.315) | (0.039) | (0.039) | (0.061) | - |
| Exposure Duration: 7 years | -0.122** | -0.195*** | -0.106* | 0.424*** | -0.146*** | -0.109** | -0.076 | - |
|  | (0.052) | (0.047) | (0.063) | (0.149) | (0.049) | (0.044) | (0.061) | - |
| Exposure Duration: 8 years | -0.022 | -0.198*** | -0.104 | 0.441*** | -0.299*** | -0.172*** | 0.004 | 0 |
|  | (0.070) | (0.055) | (0.070) | (0.150) | (0.067) | (0.048) | (0.078) | omitted |
| Exposure Duration: 9 years | -0.137* | -0.178*** | -0.134* | 0.486*** | -0.264*** | -0.210*** | -0.063 | - |
|  | (0.072) | (0.066) | (0.075) | (0.162) | (0.079) | (0.058) | (0.067) | - |
| Exposure Duration: 10 years | -0.173* | -0.144** | -0.127 | -0.358 | -0.241** | -0.127** | -0.280*** | -1.169*** |
|  | (0.104) | (0.063) | (0.098) | (0.229) | (0.111) | (0.059) | (0.083) | (0.102) |
| Exposure Duration: 11 years | -0.082 | -0.076 | -0.148 | 0.351 | -0.068 | -0.197** | -0.233** | -1.000 |
|  | (0.130) | (0.088) | (0.096) | (0.216) | (0.073) | (0.077) | (0.096) | (0.000) |
| Exposure Duration: 12 years | -0.364 | -0.120 | -0.056 | 0.351** | -0.460** | -0.275*** | -0.230** | - |
|  | (0.440) | (0.111) | (0.088) | (0.168) | (0.208) | (0.091) | (0.101) | - |
| Exposure Duration: 13 years | -0.357** | -0.213* | -0.079 | 0.430** | -0.008 | -0.241** | -0.169* | - |
|  | (0.172) | (0.112) | (0.107) | (0.209) | (0.250) | (0.098) | (0.091) | - |
| Exposure Duration: 14 years | -0.390* | -0.217* | -0.138 | - | -0.848*** | -0.322*** | -0.331*** | - |
|  | (0.222) | (0.124) | (0.132) | - | (0.109) | (0.110) | (0.107) | - |
| Exposure Duration: 15 years | -0.646* | -0.367*** | -0.124 | - | -0.690*** | -0.184 | -0.186* | -0.990*** |
|  | (0.365) | (0.115) | (0.160) | - | (0.039) | (0.116) | (0.102) | (0.083) |
|  |  |  |  |  |  |  |  |  |
| Full-time employment | Ref. | Ref. | Ref. | Ref. | Ref. | Ref. | Ref. | Ref. |
| Part-time employment | 0.017 | -0.015 | -0.034 | -0.022 | 0.069*** | -0.065 | -0.053 | -0.045 |
|  | (0.020) | (0.022) | (0.033) | (0.145) | (0.024) | (0.043) | (0.056) | (0.120) |
| No employment | 0.157*** | 0.003 | -0.405*** | -1.022*** | -0.033 | -0.136 | -0.339** | -1.002*** |
|  | (0.039) | (0.059) | (0.107) | (0.145) | (0.046) | (0.084) | (0.133) | (0.052) |
| Pensioners | 0.445*** | -0.326 | -0.215* | -0.181 | -0.614* | -0.819** | -0.203 | -0.208 |
|  | (0.028) | (0.215) | (0.110) | (0.164) | (0.358) | (0.334) | (0.159) | (0.151) |
| In education | 0.025 | 0.062 | 0.023 | 1.029*** | 0.067*** | -0.081 | -0.007 | - |
|  | (0.018) | (0.085) | (0.142) | (0.187) | (0.016) | (0.086) | (0.409) | - |
|  |  |  |  |  |  |  |  |  |
| Low income position | -0.007 | -0.001 | -0.014 | 0.078 | 0.006 | 0.004 | 0.039 | -0.016 |
|  | (0.018) | (0.020) | (0.033) | (0.093) | (0.017) | (0.027) | (0.046) | (0.138) |
| Medium income position | Ref. | Ref. | Ref. | Ref. | Ref. | Ref. | Ref. | Ref. |
| High income position | 0.021 | -0.016 | 0.057 | -0.019 | -0.012 | -0.006 | 0.000 | -0.169* |
|  | (0.019) | (0.026) | (0.035) | (0.099) | (0.016) | (0.021) | (0.034) | (0.102) |
|  |  |  |  |  |  |  |  |  |
| Single (incl. divorced) | Ref. | Ref. | Ref. | Ref. | Ref. | Ref. | Ref. | Ref. |
| Married | -0.088 | 0.098*** | 0.135** | 0 | -0.124 | -0.029 | 0.108* | 0.330* |
|  | (0.055) | (0.033) | (0.061) | omitted | (0.083) | (0.035) | (0.058) | (0.187) |
| Non-marital partnership | -0.061*** | -0.018 | 0.056 | 0.182 | -0.026* | -0.012 | 0.014 | 0.199 |
|  | (0.016) | (0.021) | (0.035) | (0.161) | (0.015) | (0.022) | (0.042) | (0.174) |
|  |  |  |  |  |  |  |  |  |
| No partner in household | Ref. | Ref. | Ref. | Ref. | Ref. | Ref. | Ref. | Ref. |
| Partner living in household | -0.022 | -0.051* | -0.124* | -0.202 | -0.028 | -0.029 | 0.056 | -0.161 |
|  | (0.020) | (0.027) | (0.068) | (0.148) | (0.021) | (0.027) | (0.043) | (0.140) |
|  |  |  |  |  |  |  |  |  |
| No children<5 in household | Ref. | Ref. | Ref. | Ref. | Ref. | Ref. | Ref. | Ref. |
| Household with children<5 | -0.049* | 0.055* | 0.028 | 0.044 | -0.006 | 0.019 | 0.024 | 0 |
|  | (0.028) | (0.033) | (0.107) | (0.100) | (0.030) | (0.032) | (0.179) | omitted |
|  |  |  |  |  |  |  |  |  |
| Western Germany | Ref. | Ref. | Ref. | Ref. | Ref. | Ref. | Ref. | Ref. |
| Eastern Germany | -0.027 | 0.151** | 0.009 | 0 | -0.002 | 0.078 | 0.160* | 0 |
|  | (0.049) | (0.069) | (0.126) | omitted | (0.054) | (0.127) | (0.087) | omitted |
|  |  |  |  |  |  |  |  |  |
| Constant | 2.891*** | 2.551*** | 2.280*** | 2.280*** | 2.983*** | 2.678*** | 2.292*** | 2.379*** |
|  | (0.020) | (0.025) | (0.036) | (0.127) | (0.019) | (0.040) | (0.034) | (0.137) |
|  |  |  |  |  |  |  |  |  |
| Observations | 22,824 | 20,273 | 9,974 | 829 | 24,639 | 17,184 | 6,987 | 562 |

German Socio-Economic Panel 1994-2017; Significance levels are *p<0.05, **p<0.01 and ***p<0.001; Corresponds to Figure 4b

Shown are unstandardized beta coefficients (β) and panel robust standard errors (SE)

**App. Table 10** Predictive margins on changes in SRH for each year of continuing exposure towards occupational worries in different life stages

|  | Women | | | | Men | | | |
| --- | --- | --- | --- | --- | --- | --- | --- | --- |
| Predictive margins | Emerging Adulthood | Early Middle Adulthood | Later Middle Adulthood | Late  Adulthood | Emerging Adulthood | Early Middle Adulthood | Later Middle Adulthood | Late  Adulthood |
| No exposure | Ref. | Ref. | Ref. | Ref. | Ref. | Ref. | Ref. | Ref. |
| Exposure dur.: 1 yr | -0.046*** | -0.050*** | 0.009 | 0.044 | -0.025* | -0.053*** | -0.044 | Not estimable |
|  | (-0.072 - -0.019) | (-0.082 - -0.017) | (-0.038 - 0.057) | (-0.152 - 0.240) | (-0.051 - 0.000) | (-0.086 - -0.020) | (-0.098 - 0.010) |  |
| Exposure dur.: 2 yrs | -0.060*** | -0.059*** | 0.020 | 0.212 | -0.077*** | -0.055*** | -0.075** | Not estimable |
|  | (-0.095 - -0.026) | (-0.100 - -0.019) | (-0.043 - 0.084) | (-0.127 - 0.551) | (-0.111 - -0.044) | (-0.097 - -0.013) | (-0.142 - -0.008) |  |
| Exposure dur.: 3 yrs | -0.054** | -0.093*** | -0.025 | 0.388** | -0.136*** | -0.080*** | -0.052 | Not estimable |
|  | (-0.098 - -0.010) | (-0.143 - -0.043) | (-0.103 - 0.053) | (0.054 - 0.721) | (-0.179 - -0.092) | (-0.131 - -0.030) | (-0.135 - 0.032) |  |
| Exposure dur.: 4 yrs | -0.075*** | -0.118*** | -0.081* | 0.680** | -0.136*** | -0.082*** | -0.066 | Not estimable |
|  | (-0.130 - -0.021) | (-0.174 - -0.063) | (-0.172 - 0.010) | (0.101 - 1.258) | (-0.191 - -0.082) | (-0.140 - -0.023) | (-0.163 - 0.031) |  |
| Exposure dur.: 5 yrs | -0.067* | -0.138*** | -0.072 | 0.864 | -0.104*** | -0.093*** | -0.005 | Not estimable |
|  | (-0.137 - 0.003) | (-0.202 - -0.073) | (-0.166 - 0.022) | (-0.418 - 2.146) | (-0.171 - -0.038) | (-0.161 - -0.024) | (-0.109 - 0.098) |  |
| Exposure dur.: 6 yrs | -0.093** | -0.178*** | -0.054 | 0.436 | -0.203*** | -0.115*** | -0.003 | Not estimable |
|  | (-0.174 - -0.012) | (-0.258 - -0.098) | (-0.166 - 0.058) | (-0.182 - 1.055) | (-0.280 - -0.126) | (-0.192 - -0.038) | (-0.123 - 0.116) |  |
| Exposure dur.: 7 yrs | -0.122** | -0.195*** | -0.106* | 0.424*** | -0.146*** | -0.109** | -0.076 | Not estimable |
|  | (-0.224 - -0.020) | (-0.287 - -0.103) | (-0.230 - 0.018) | (0.132 - 0.717) | (-0.242 - -0.050) | (-0.195 - -0.023) | (-0.196 - 0.043) |  |
| Exposure dur.: 8 yrs | -0.022 | -0.198*** | -0.104 | 0.441*** | -0.299*** | -0.172*** | 0.004 | Not estimable |
|  | (-0.160 - 0.116) | (-0.306 - -0.090) | (-0.240 - 0.033) | (0.148 - 0.734) | (-0.430 - -0.168) | (-0.266 - -0.078) | (-0.149 - 0.156) |  |
| Exposure dur.: 9 yrs | -0.137* | -0.178*** | -0.134* | 0.486*** | -0.264*** | -0.210*** | -0.063 | Not estimable |
|  | (-0.279 - 0.005) | (-0.308 - -0.049) | (-0.282 - 0.013) | (0.167 - 0.804) | (-0.419 - -0.108) | (-0.324 - -0.096) | (-0.195 - 0.069) |  |
| Exposure dur.: 10 yrs | -0.173* | -0.144** | -0.127 | -0.358 | -0.241** | -0.127** | -0.280*** | Not estimable |
|  | (-0.377 - 0.030) | (-0.266 - -0.021) | (-0.319 - 0.066) | (-0.806 - 0.090) | (-0.459 - -0.022) | (-0.243 - -0.011) | (-0.444 - -0.117) |  |
| Exposure dur.: 11 yrs | -0.082 | -0.076 | -0.148 | 0.351 | -0.068 | -0.197** | -0.233** | Not estimable |
|  | (-0.337 - 0.173) | (-0.249 - 0.097) | (-0.336 - 0.041) | (-0.072 - 0.774) | (-0.212 - 0.076) | (-0.348 - -0.046) | (-0.421 - -0.045) |  |
| Exposure dur.: 12 yrs | -0.364 | -0.120 | -0.056 | 0.351** | -0.460** | -0.275*** | -0.230** | Not estimable |
|  | (-1.226 - 0.498) | (-0.338 - 0.098) | (-0.229 - 0.116) | (0.021 - 0.681) | (-0.866 - -0.053) | (-0.452 - -0.097) | (-0.428 - -0.031) |  |
| Exposure dur.: 14 yrs | -0.357** | -0.213* | -0.079 | 0.430** | -0.008 | -0.241** | -0.169* | Not estimable |
|  | (-0.694 - -0.020) | (-0.432 - 0.006) | (-0.288 - 0.131) | (0.020 - 0.840) | (-0.497 - 0.482) | (-0.432 - -0.050) | (-0.348 - 0.010) |  |
| Exposure dur.: 14 yrs | -0.390* | -0.217* | -0.138 | - | -0.848*** | -0.322*** | -0.331*** | Not estimable |
|  | (-0.826 - 0.045) | (-0.461 - 0.027) | (-0.397 - 0.122) | - | (-1.061 - -0.634) | (-0.537 - -0.107) | (-0.540 - -0.122) |  |
| Exposure dur.: 15 yrs | -0.646* | -0.367*** | -0.124 | - | -0.690*** | -0.184 | -0.186* | Not estimable |
|  | (-1.362 - 0.069) | (-0.593 - -0.141) | (-0.438 - 0.191) | - | (-0.765 - -0.614) | (-0.411 - 0.042) | (-0.386 - 0.014) |  |
|  |  |  |  |  |  |  |  |  |
| Observations | 22,824 | 20,273 | 9,974 | 829 | 24,639 | 17,184 | 6,987 | 562 |

SRH - Self-rated Health; German Socio-Economic Panel 1994-2017; Significance levels are *p<0.05, **p<0.01 and ***p<0.001; Corresponds to Figure 4b

Shown are the average marginal effects (AME) for the specific exposure duration and the 95% confidence intervals as calculated from panel robust standard errors (SE)

**App. Table 11** Fixed-effects regressions for impact of continuing exposure to below-average housing satisfaction on SRH in different stages (regression outputs)

|  | Women | | | | Men | | | |
| --- | --- | --- | --- | --- | --- | --- | --- | --- |
| Impact on Self-rated health | Emerging Adulthood | Early Middle Adulthood | Later Middle Adulthood | Late Adulthood | Emerging Adulthood | Early Middle Adulthood | Later Middle Adulthood | Late Adulthood |
| No exposure | Ref. | Ref. | Ref. | Ref. | Ref. | Ref. | Ref. | Ref. |
|  |  |  |  |  |  |  |  |  |
| Exposure Duration: 1 year | -0.084*** | -0.054*** | -0.077*** | -0.019 | -0.072*** | -0.032** | -0.073*** | -0.085*** |
|  | (0.011) | (0.014) | (0.019) | (0.021) | (0.010) | (0.015) | (0.022) | (0.031) |
| Exposure Duration: 2 years | -0.080*** | -0.071*** | -0.103*** | -0.026 | -0.097*** | -0.083*** | -0.076** | -0.083* |
|  | (0.015) | (0.019) | (0.026) | (0.031) | (0.015) | (0.020) | (0.031) | (0.046) |
| Exposure Duration: 3 years | -0.112*** | -0.066*** | -0.085*** | -0.015 | -0.144*** | -0.105*** | -0.126*** | -0.137** |
|  | (0.019) | (0.023) | (0.030) | (0.040) | (0.019) | (0.025) | (0.037) | (0.063) |
| Exposure Duration: 4 years | -0.096*** | -0.106*** | -0.053 | -0.061 | -0.143*** | -0.090*** | -0.104** | -0.096 |
|  | (0.026) | (0.027) | (0.037) | (0.053) | (0.024) | (0.030) | (0.042) | (0.071) |
| Exposure Duration: 5 years | -0.142*** | -0.130*** | -0.152*** | -0.098* | -0.148*** | -0.162*** | -0.024 | 0.041 |
|  | (0.035) | (0.032) | (0.046) | (0.057) | (0.031) | (0.036) | (0.050) | (0.081) |
| Exposure Duration: 6 years | -0.176*** | -0.163*** | -0.129** | -0.110* | -0.212*** | -0.157*** | -0.091 | 0.122 |
|  | (0.040) | (0.037) | (0.054) | (0.066) | (0.039) | (0.038) | (0.056) | (0.087) |
| Exposure Duration: 7 years | -0.114** | -0.208*** | -0.179*** | -0.196** | -0.243*** | -0.143*** | -0.112 | 0.056 |
|  | (0.051) | (0.042) | (0.056) | (0.083) | (0.048) | (0.045) | (0.071) | (0.106) |
| Exposure Duration: 8 years | -0.226*** | -0.108** | -0.215*** | -0.060 | -0.203*** | -0.233*** | -0.132** | -0.103 |
|  | (0.074) | (0.051) | (0.068) | (0.072) | (0.057) | (0.052) | (0.067) | (0.091) |
| Exposure Duration: 9 years | -0.051 | -0.233*** | -0.089 | -0.287*** | -0.105 | -0.217*** | -0.190** | -0.127 |
|  | (0.089) | (0.056) | (0.073) | (0.103) | (0.071) | (0.059) | (0.079) | (0.113) |
| Exposure Duration: 10 years | -0.142 | -0.207*** | -0.112 | -0.174 | 0.059 | -0.150** | -0.111 | 0.041 |
|  | (0.125) | (0.070) | (0.075) | (0.108) | (0.103) | (0.058) | (0.085) | (0.158) |
| Exposure Duration: 11 years | -0.229 | -0.290*** | -0.195** | -0.204* | -0.045 | -0.258*** | -0.247*** | -0.104 |
|  | (0.171) | (0.084) | (0.095) | (0.105) | (0.103) | (0.070) | (0.090) | (0.154) |
| Exposure Duration: 12 years | -0.568** | -0.203** | -0.155 | -0.205* | -0.460*** | -0.297*** | -0.349*** | -0.076 |
|  | (0.269) | (0.095) | (0.102) | (0.107) | (0.160) | (0.087) | (0.100) | (0.135) |
| Exposure Duration: 13 years | -1.251*** | -0.385*** | -0.148 | -0.086 | -0.038 | -0.348*** | -0.434*** | -0.225 |
|  | (0.370) | (0.116) | (0.106) | (0.129) | (0.238) | (0.089) | (0.131) | (0.174) |
| Exposure Duration: 14 years | -1.758** | -0.305** | -0.297*** | -0.058 | -0.033 | -0.457*** | -0.406*** | -0.348** |
|  | (0.838) | (0.123) | (0.106) | (0.171) | (0.191) | (0.095) | (0.127) | (0.175) |
| Exposure Duration: 15 years | -0.467*** | -0.437*** | -0.287* | -0.303* | - | -0.435*** | -0.461*** | -0.329* |
|  | (0.069) | (0.129) | (0.151) | (0.167) | - | (0.108) | (0.165) | (0.195) |
| Full-time employment | Ref. | Ref. | Ref. | Ref. | Ref. | Ref. | Ref. | Ref. |
| Part-time employment | 0.020 | -0.040** | -0.029 | 0.123 | 0.071*** | -0.091** | -0.035 | 0.011 |
|  | (0.017) | (0.020) | (0.029) | (0.126) | (0.019) | (0.038) | (0.048) | (0.087) |
| No employment | 0.071*** | -0.095*** | -0.158*** | -0.036 | 0.003 | -0.200*** | -0.173*** | -0.430*** |
|  | (0.019) | (0.026) | (0.039) | (0.160) | (0.018) | (0.033) | (0.042) | (0.152) |
| Pensioners | -0.027 | -0.360*** | -0.097** | -0.060 | -0.380 | -0.340*** | -0.024 | -0.221*** |
|  | (0.262) | (0.083) | (0.039) | (0.129) | (0.279) | (0.104) | (0.050) | (0.079) |
| In education | 0.043*** | -0.001 | 0.127 | 0.192 | 0.083*** | -0.068 | -0.227 | 0.146 |
|  | (0.014) | (0.062) | (0.108) | (0.178) | (0.013) | (0.057) | (0.188) | (0.234) |
|  |  |  |  |  |  |  |  |  |
| Low income position | -0.011 | -0.004 | -0.055** | -0.008 | 0.006 | -0.027 | -0.117*** | -0.065 |
|  | (0.013) | (0.018) | (0.024) | (0.029) | (0.014) | (0.025) | (0.034) | (0.047) |
| Medium income position | Ref. | Ref. | Ref. | Ref. | Ref. | Ref. | Ref. | Ref. |
| High income position | 0.022 | -0.028 | 0.068** | 0.032 | -0.000 | -0.030 | 0.029 | 0.033 |
|  | (0.016) | (0.025) | (0.032) | (0.038) | (0.014) | (0.021) | (0.030) | (0.048) |
|  |  |  |  |  |  |  |  |  |
| Single (incl. divorced) | Ref. | Ref. | Ref. | Ref. | Ref. | Ref. | Ref. | Ref. |
| Married | -0.123*** | 0.095*** | 0.142*** | -0.027 | -0.064 | 0.007 | 0.093* | 0.082 |
|  | (0.044) | (0.030) | (0.051) | (0.084) | (0.077) | (0.034) | (0.051) | (0.114) |
| Non-marital partnership | -0.057*** | -0.005 | 0.062** | 0.087* | -0.027** | -0.007 | 0.051 | -0.006 |
|  | (0.012) | (0.019) | (0.028) | (0.053) | (0.012) | (0.022) | (0.036) | (0.050) |
|  |  |  |  |  |  |  |  |  |
| No partner in household | Ref. | Ref. | Ref. | Ref. | Ref. | Ref. | Ref. | Ref. |
| Partner living in household | -0.013 | -0.021 | -0.118** | 0.037 | -0.033* | -0.045* | 0.021 | -0.052 |
|  | (0.017) | (0.024) | (0.053) | (0.097) | (0.018) | (0.027) | (0.041) | (0.084) |
|  |  |  |  |  |  |  |  |  |
| No children<5 in household | Ref. | Ref. | Ref. | Ref. | Ref. | Ref. | Ref. | Ref. |
| Household with children<5 | 0.018 | 0.095*** | 0.097 | -0.068 | 0.013 | 0.034 | 0.059 | 1.052** |
|  | (0.021) | (0.027) | (0.092) | (0.097) | (0.026) | (0.030) | (0.119) | (0.445) |
|  |  |  |  |  |  |  |  |  |
| Western Germany | Ref. | Ref. | Ref. | Ref. | Ref. | Ref. | Ref. | Ref. |
| Eastern Germany | -0.016 | 0.126* | -0.070 | 0.239* | 0.006 | -0.017 | 0.162 | -0.158 |
|  | (0.038) | (0.068) | (0.091) | (0.144) | (0.042) | (0.122) | (0.102) | (0.319) |
|  |  |  |  |  |  |  |  |  |
| Constant | 2.874*** | 2.467*** | 2.197*** | 1.904*** | 2.975*** | 2.648*** | 2.159*** | 2.307*** |
|  | (0.017) | (0.025) | (0.033) | (0.133) | (0.016) | (0.039) | (0.039) | (0.115) |
|  |  |  |  |  |  |  |  |  |
| Observations | 36,265 | 25,749 | 15,623 | 11,104 | 36,279 | 20,373 | 10,572 | 4,992 |

German Socio-Economic Panel 1994-2017; Significance levels are *p<0.05, **p<0.01 and ***p<0.001; Corresponds to Figure 5a

Shown are unstandardized beta coefficients (β) and panel robust standard errors (SE)

**App. Table 12** Predictive margins on changes in SRH for each year of continuing exposure towards below-average housing satisfaction in different life stages

|  | Women | | | | Men | | | |
| --- | --- | --- | --- | --- | --- | --- | --- | --- |
| Predictive margins | Emerging Adulthood | Early Middle Adulthood | Later Middle Adulthood | Late  Adulthood | Emerging Adulthood | Early Middle Adulthood | Later Middle Adulthood | Late  Adulthood |
| No exposure | Ref. | Ref. | Ref. | Ref. | Ref. | Ref. | Ref. | Ref. |
|  |  |  |  |  |  |  |  |  |
| Exposure dur.: 1 yr | -0.084*** | -0.054*** | -0.077*** | -0.019 | -0.072*** | -0.032** | -0.073*** | -0.085*** |
|  | (-0.105 - -0.063) | (-0.082 - -0.026) | (-0.114 - -0.040) | (-0.059 - 0.021) | (-0.092 - -0.052) | (-0.062 - -0.002) | (-0.115 - -0.031) | (-0.146 - -0.025) |
| Exposure dur.: 2 yrs | -0.080*** | -0.071*** | -0.103*** | -0.026 | -0.097*** | -0.083*** | -0.076** | -0.083* |
|  | (-0.110 - -0.051) | (-0.109 - -0.034) | (-0.154 - -0.051) | (-0.087 - 0.035) | (-0.125 - -0.068) | (-0.122 - -0.043) | (-0.137 - -0.016) | (-0.174 - 0.007) |
| Exposure dur.: 3 yrs | -0.112*** | -0.066*** | -0.085*** | -0.015 | -0.144*** | -0.105*** | -0.126*** | -0.137** |
|  | (-0.150 - -0.074) | (-0.111 - -0.020) | (-0.143 - -0.027) | (-0.093 - 0.063) | (-0.181 - -0.107) | (-0.153 - -0.056) | (-0.197 - -0.054) | (-0.260 - -0.014) |
| Exposure dur.: 4 yrs | -0.096*** | -0.106*** | -0.053 | -0.061 | -0.143*** | -0.090*** | -0.104** | -0.096 |
|  | (-0.146 - -0.046) | (-0.160 - -0.053) | (-0.125 - 0.018) | (-0.165 - 0.043) | (-0.190 - -0.095) | (-0.149 - -0.030) | (-0.185 - -0.022) | (-0.236 - 0.044) |
| Exposure dur.: 5 yrs | -0.142*** | -0.130*** | -0.152*** | -0.098* | -0.148*** | -0.162*** | -0.024 | 0.041 |
|  | (-0.211 - -0.073) | (-0.193 - -0.068) | (-0.243 - -0.061) | (-0.209 - 0.013) | (-0.208 - -0.088) | (-0.233 - -0.091) | (-0.121 - 0.074) | (-0.117 - 0.199) |
| Exposure dur.: 6 yrs | -0.176*** | -0.163*** | -0.129** | -0.110* | -0.212*** | -0.157*** | -0.091 | 0.122 |
|  | (-0.255 - -0.097) | (-0.236 - -0.090) | (-0.235 - -0.024) | (-0.238 - 0.019) | (-0.288 - -0.136) | (-0.232 - -0.081) | (-0.200 - 0.018) | (-0.047 - 0.292) |
| Exposure dur.: 7 yrs | -0.114** | -0.208*** | -0.179*** | -0.196** | -0.243*** | -0.143*** | -0.112 | 0.056 |
|  | (-0.214 - -0.013) | (-0.289 - -0.126) | (-0.289 - -0.069) | (-0.359 - -0.033) | (-0.338 - -0.149) | (-0.232 - -0.055) | (-0.250 - 0.026) | (-0.151 - 0.263) |
| Exposure dur.: 8 yrs | -0.226*** | -0.108** | -0.215*** | -0.060 | -0.203*** | -0.233*** | -0.132** | -0.103 |
|  | (-0.370 - -0.081) | (-0.207 - -0.008) | (-0.349 - -0.081) | (-0.201 - 0.080) | (-0.315 - -0.092) | (-0.334 - -0.132) | (-0.263 - -0.001) | (-0.281 - 0.075) |
| Exposure dur.: 9 yrs | -0.051 | -0.233*** | -0.089 | -0.287*** | -0.105 | -0.217*** | -0.190** | -0.127 |
|  | (-0.226 - 0.123) | (-0.344 - -0.123) | (-0.232 - 0.053) | (-0.489 - -0.084) | (-0.245 - 0.034) | (-0.333 - -0.102) | (-0.344 - -0.035) | (-0.349 - 0.095) |
| Exposure dur.: 10 yrs | -0.142 | -0.207*** | -0.112 | -0.174 | 0.059 | -0.150** | -0.111 | 0.041 |
|  | (-0.387 - 0.103) | (-0.344 - -0.071) | (-0.258 - 0.035) | (-0.386 - 0.037) | (-0.142 - 0.261) | (-0.264 - -0.035) | (-0.277 - 0.055) | (-0.269 - 0.350) |
| Exposure dur.: 11 yrs | -0.229 | -0.290*** | -0.195** | -0.204* | -0.045 | -0.258*** | -0.247*** | -0.104 |
|  | (-0.565 - 0.107) | (-0.454 - -0.127) | (-0.381 - -0.009) | (-0.411 - 0.002) | (-0.247 - 0.157) | (-0.395 - -0.122) | (-0.423 - -0.072) | (-0.405 - 0.197) |
| Exposure dur.: 12 yrs | -0.568** | -0.203** | -0.155 | -0.205* | -0.460*** | -0.297*** | -0.349*** | -0.076 |
|  | (-1.095 - -0.040) | (-0.390 - -0.016) | (-0.356 - 0.045) | (-0.414 - 0.004) | (-0.773 - -0.147) | (-0.467 - -0.126) | (-0.546 - -0.152) | (-0.341 - 0.189) |
| Exposure dur.: 14 yrs | -1.251*** | -0.385*** | -0.148 | -0.086 | -0.038 | -0.348*** | -0.434*** | -0.225 |
|  | (-1.976 - -0.525) | (-0.612 - -0.158) | (-0.356 - 0.061) | (-0.338 - 0.166) | (-0.506 - 0.429) | (-0.522 - -0.174) | (-0.691 - -0.177) | (-0.567 - 0.117) |
| Exposure dur.: 14 yrs | -1.758** | -0.305** | -0.297*** | -0.058 | -0.033 | -0.457*** | -0.406*** | -0.348** |
|  | (-3.400 - -0.116) | (-0.547 - -0.063) | (-0.505 - -0.089) | (-0.394 - 0.278) | (-0.407 - 0.342) | (-0.644 - -0.271) | (-0.655 - -0.156) | (-0.692 - -0.005) |
| Exposure dur.: 15 yrs | -0.467*** | -0.437*** | -0.287* | -0.303* | - | -0.435*** | -0.461*** | -0.329* |
|  | (-0.603 - -0.331) | (-0.689 - -0.185) | (-0.584 - 0.010) | (-0.630 - 0.023) | - | (-0.647 - -0.222) | (-0.785 - -0.137) | (-0.711 - 0.053) |
|  |  |  |  |  |  |  |  |  |
| Observations | 36,265 | 25,749 | 15,623 | 11,104 | 36,279 | 20,373 | 10,572 | 4,992 |

SRH - Self-rated Health; German Socio-Economic Panel 1994-2017; Significance levels are *p<0.05, **p<0.01 and ***p<0.001; Corresponds to Figure 5a

Shown are the average marginal effects (AME) for the specific exposure duration and the 95% confidence intervals as calculated from panel robust standard errors (SE)

**App. Table 13** Fixed-effects regressions for impact of continuing exposure to below-average income satisfaction on SRH in different stages (regression outputs)

|  | Women | | | | Men | | | |
| --- | --- | --- | --- | --- | --- | --- | --- | --- |
| Impact on Self-rated health | Emerging Adulthood | Early Middle Adulthood | Later Middle Adulthood | Late Adulthood | Emerging Adulthood | Early Middle Adulthood | Later Middle Adulthood | Late Adulthood |
| No exposure | Ref. | Ref. | Ref. | Ref. | Ref. | Ref. | Ref. | Ref. |
|  |  |  |  |  |  |  |  |  |
| Exposure Duration: 1 year | -0.065*** | -0.075*** | -0.104*** | -0.028 | -0.069*** | -0.052*** | -0.070*** | -0.099*** |
|  | (0.011) | (0.015) | (0.019) | (0.022) | (0.011) | (0.016) | (0.024) | (0.034) |
| Exposure Duration: 2 years | -0.093*** | -0.103*** | -0.113*** | -0.038 | -0.116*** | -0.099*** | -0.116*** | -0.144*** |
|  | (0.015) | (0.020) | (0.023) | (0.028) | (0.014) | (0.022) | (0.030) | (0.044) |
| Exposure Duration: 3 years | -0.115*** | -0.110*** | -0.121*** | -0.039 | -0.136*** | -0.141*** | -0.121*** | -0.093* |
|  | (0.018) | (0.023) | (0.029) | (0.034) | (0.018) | (0.025) | (0.033) | (0.051) |
| Exposure Duration: 4 years | -0.118*** | -0.166*** | -0.157*** | -0.052 | -0.166*** | -0.135*** | -0.144*** | -0.096 |
|  | (0.022) | (0.027) | (0.032) | (0.039) | (0.023) | (0.029) | (0.042) | (0.061) |
| Exposure Duration: 5 years | -0.154*** | -0.203*** | -0.147*** | -0.098** | -0.168*** | -0.183*** | -0.157*** | -0.193*** |
|  | (0.029) | (0.029) | (0.034) | (0.043) | (0.028) | (0.033) | (0.042) | (0.068) |
| Exposure Duration: 6 years | -0.157*** | -0.173*** | -0.172*** | -0.091* | -0.219*** | -0.209*** | -0.205*** | -0.123 |
|  | (0.033) | (0.034) | (0.039) | (0.048) | (0.034) | (0.038) | (0.047) | (0.079) |
| Exposure Duration: 7 years | -0.160*** | -0.251*** | -0.214*** | -0.156*** | -0.201*** | -0.201*** | -0.203*** | -0.080 |
|  | (0.041) | (0.039) | (0.042) | (0.054) | (0.042) | (0.041) | (0.058) | (0.090) |
| Exposure Duration: 8 years | -0.128** | -0.204*** | -0.251*** | -0.090 | -0.200*** | -0.235*** | -0.185*** | -0.075 |
|  | (0.050) | (0.041) | (0.048) | (0.062) | (0.055) | (0.048) | (0.062) | (0.098) |
| Exposure Duration: 9 years | -0.116* | -0.259*** | -0.167*** | -0.183*** | -0.212*** | -0.267*** | -0.260*** | -0.233** |
|  | (0.064) | (0.047) | (0.056) | (0.067) | (0.064) | (0.051) | (0.062) | (0.101) |
| Exposure Duration: 10 years | -0.103 | -0.225*** | -0.089 | -0.174** | -0.213** | -0.207*** | -0.248*** | -0.206** |
|  | (0.095) | (0.049) | (0.061) | (0.072) | (0.084) | (0.055) | (0.067) | (0.104) |
| Exposure Duration: 11 years | -0.023 | -0.282*** | -0.249*** | -0.182** | -0.127 | -0.196*** | -0.289*** | -0.257** |
|  | (0.100) | (0.060) | (0.064) | (0.087) | (0.088) | (0.060) | (0.077) | (0.123) |
| Exposure Duration: 12 years | -0.034 | -0.258*** | -0.269*** | -0.206** | -0.477*** | -0.295*** | -0.303*** | -0.293** |
|  | (0.141) | (0.067) | (0.068) | (0.092) | (0.099) | (0.069) | (0.091) | (0.142) |
| Exposure Duration: 13 years | -0.406*** | -0.250*** | -0.281*** | -0.179** | -0.339* | -0.262*** | -0.385*** | -0.264 |
|  | (0.155) | (0.079) | (0.079) | (0.086) | (0.182) | (0.086) | (0.109) | (0.161) |
| Exposure Duration: 14 years | -0.535* | -0.291*** | -0.330*** | -0.030 | -0.513** | -0.525*** | -0.313*** | -0.236 |
|  | (0.274) | (0.076) | (0.098) | (0.097) | (0.210) | (0.094) | (0.112) | (0.192) |
| Exposure Duration: 15 years | -0.093 | -0.373*** | -0.141 | -0.252** | -0.710 | -0.380*** | -0.355*** | -0.120 |
|  | (0.119) | (0.111) | (0.099) | (0.123) | (1.057) | (0.103) | (0.127) | (0.174) |
| Full-time employment | Ref. | Ref. | Ref. | Ref. | Ref. | Ref. | Ref. | Ref. |
| Part-time employment | 0.024 | -0.025 | -0.015 | 0.139 | 0.078*** | -0.071* | -0.012 | 0.044 |
|  | (0.017) | (0.021) | (0.030) | (0.125) | (0.019) | (0.038) | (0.047) | (0.086) |
| No employment | 0.080*** | -0.073*** | -0.135*** | -0.015 | 0.018 | -0.184*** | -0.151*** | -0.354** |
|  | (0.019) | (0.026) | (0.039) | (0.160) | (0.018) | (0.032) | (0.042) | (0.162) |
| Pensioners | -0.026 | -0.301*** | -0.086** | -0.049 | -0.338 | -0.313*** | -0.013 | -0.184** |
|  | (0.259) | (0.081) | (0.039) | (0.128) | (0.277) | (0.103) | (0.050) | (0.079) |
| In education | 0.046*** | 0.007 | 0.141 | 0.231 | 0.087*** | -0.050 | -0.144 | 0.184 |
|  | (0.014) | (0.061) | (0.109) | (0.183) | (0.013) | (0.057) | (0.181) | (0.230) |
|  |  |  |  |  |  |  |  |  |
| Low income position | 0.001 | 0.015 | -0.040* | -0.002 | 0.017 | -0.013 | -0.105*** | -0.061 |
|  | (0.013) | (0.018) | (0.024) | (0.029) | (0.014) | (0.025) | (0.034) | (0.048) |
| Medium income position | Ref. | Ref. | Ref. | Ref. | Ref. | Ref. | Ref. | Ref. |
| High income position | 0.014 | -0.038 | 0.052 | 0.030 | -0.010 | -0.040* | 0.015 | 0.014 |
|  | (0.016) | (0.024) | (0.032) | (0.038) | (0.014) | (0.021) | (0.030) | (0.048) |
|  |  |  |  |  |  |  |  |  |
| Single (incl. divorced) | Ref. | Ref. | Ref. | Ref. | Ref. | Ref. | Ref. | Ref. |
| Married | -0.120*** | 0.083*** | 0.141*** | -0.022 | -0.058 | 0.005 | 0.100* | 0.119 |
|  | (0.044) | (0.030) | (0.051) | (0.087) | (0.078) | (0.033) | (0.052) | (0.121) |
| Non-marital partnership | -0.057*** | -0.007 | 0.050* | 0.091* | -0.028** | -0.000 | 0.067* | -0.006 |
|  | (0.012) | (0.019) | (0.028) | (0.053) | (0.012) | (0.022) | (0.036) | (0.050) |
|  |  |  |  |  |  |  |  |  |
| No partner in household | Ref. | Ref. | Ref. | Ref. | Ref. | Ref. | Ref. | Ref. |
| Partner living in household | -0.013 | -0.032 | -0.119** | 0.013 | -0.026 | -0.044 | 0.019 | -0.055 |
|  | (0.017) | (0.024) | (0.054) | (0.096) | (0.018) | (0.027) | (0.041) | (0.082) |
|  |  |  |  |  |  |  |  |  |
| No children<5 in household | Ref. | Ref. | Ref. | Ref. | Ref. | Ref. | Ref. | Ref. |
| Household with children<5 | 0.020 | 0.090*** | 0.066 | -0.093 | 0.009 | 0.034 | 0.062 | 1.048** |
|  | (0.021) | (0.027) | (0.091) | (0.104) | (0.026) | (0.030) | (0.120) | (0.460) |
|  |  |  |  |  |  |  |  |  |
| Western Germany | Ref. | Ref. | Ref. | Ref. | Ref. | Ref. | Ref. | Ref. |
| Eastern Germany | -0.019 | 0.121* | -0.035 | 0.217 | 0.011 | -0.009 | 0.174* | -0.130 |
|  | (0.038) | (0.066) | (0.090) | (0.146) | (0.043) | (0.121) | (0.097) | (0.362) |
|  |  |  |  |  |  |  |  |  |
| Constant | 2.875*** | 2.496*** | 2.225*** | 1.915*** | 2.977*** | 2.663*** | 2.184*** | 2.298*** |
|  | (0.017) | (0.025) | (0.033) | (0.132) | (0.016) | (0.039) | (0.038) | (0.122) |
|  |  |  |  |  |  |  |  |  |
| Observations | 36,265 | 25,642 | 15,459 | 11,048 | 36,279 | 20,336 | 10,482 | 4,955 |

German Socio-Economic Panel 1994-2017; Significance levels are *p<0.05, **p<0.01 and ***p<0.001; Corresponds to Figure 5b

Shown are unstandardized beta coefficients (β) and panel robust standard errors (SE)

**App. Table 14** Predictive margins on changes in SRH for each year of continuing exposure towards below-average income satisfaction in different life stages

|  | Women | | | | | Men | | | | |  |
| --- | --- | --- | --- | --- | --- | --- | --- | --- | --- | --- | --- |
| Predictive margins | | Emerging Adulthood | Early Middle Adulthood | Later Middle Adulthood | Late  Adulthood | | Emerging Adulthood | Early Middle Adulthood | Later Middle Adulthood | Late  Adulthood | |
| No exposure | | Ref. | Ref. | Ref. | Ref. | | Ref. | Ref. | Ref. | Ref. | |
|  | |  |  |  |  | |  |  |  |  | |
| Exposure dur.: 1 yr | | -0.065*** | -0.075*** | -0.104*** | -0.028 | | -0.069*** | -0.052*** | -0.070*** | -0.099*** | |
|  | | (-0.087 - -0.043) | (-0.105 - -0.045) | (-0.141 - -0.067) | (-0.071 - 0.014) | | (-0.090 - -0.048) | (-0.083 - -0.020) | (-0.116 - -0.023) | (-0.165 - -0.034) | |
| Exposure dur.: 2 yrs | | -0.093*** | -0.103*** | -0.113*** | -0.038 | | -0.116*** | -0.099*** | -0.116*** | -0.144*** | |
|  | | (-0.122 - -0.064) | (-0.141 - -0.064) | (-0.159 - -0.067) | (-0.093 - 0.018) | | (-0.144 - -0.088) | (-0.142 - -0.056) | (-0.175 - -0.057) | (-0.231 - -0.058) | |
| Exposure dur.: 3 yrs | | -0.115*** | -0.110*** | -0.121*** | -0.039 | | -0.136*** | -0.141*** | -0.121*** | -0.093* | |
|  | | (-0.151 - -0.078) | (-0.154 - -0.065) | (-0.177 - -0.064) | (-0.105 - 0.027) | | (-0.172 - -0.101) | (-0.191 - -0.091) | (-0.186 - -0.056) | (-0.193 - 0.006) | |
| Exposure dur.: 4 yrs | | -0.118*** | -0.166*** | -0.157*** | -0.052 | | -0.166*** | -0.135*** | -0.144*** | -0.096 | |
|  | | (-0.161 - -0.074) | (-0.218 - -0.114) | (-0.218 - -0.095) | (-0.129 - 0.025) | | (-0.211 - -0.122) | (-0.192 - -0.079) | (-0.225 - -0.062) | (-0.216 - 0.025) | |
| Exposure dur.: 5 yrs | | -0.154*** | -0.203*** | -0.147*** | -0.098** | | -0.168*** | -0.183*** | -0.157*** | -0.193*** | |
|  | | (-0.210 - -0.097) | (-0.260 - -0.147) | (-0.213 - -0.080) | (-0.182 - -0.014) | | (-0.223 - -0.114) | (-0.248 - -0.117) | (-0.239 - -0.075) | (-0.326 - -0.060) | |
| Exposure dur.: 6 yrs | | -0.157*** | -0.173*** | -0.172*** | -0.091* | | -0.219*** | -0.209*** | -0.205*** | -0.123 | |
|  | | (-0.222 - -0.092) | (-0.240 - -0.106) | (-0.248 - -0.096) | (-0.186 - 0.003) | | (-0.286 - -0.152) | (-0.283 - -0.135) | (-0.297 - -0.112) | (-0.278 - 0.033) | |
| Exposure dur.: 7 yrs | | -0.160*** | -0.251*** | -0.214*** | -0.156*** | | -0.201*** | -0.201*** | -0.203*** | -0.080 | |
|  | | (-0.241 - -0.079) | (-0.328 - -0.175) | (-0.295 - -0.132) | (-0.263 - -0.050) | | (-0.283 - -0.119) | (-0.282 - -0.121) | (-0.317 - -0.090) | (-0.257 - 0.097) | |
| Exposure dur.: 8 yrs | | -0.128** | -0.204*** | -0.251*** | -0.090 | | -0.200*** | -0.235*** | -0.185*** | -0.075 | |
|  | | (-0.225 - -0.030) | (-0.285 - -0.123) | (-0.345 - -0.156) | (-0.212 - 0.032) | | (-0.308 - -0.091) | (-0.329 - -0.141) | (-0.306 - -0.063) | (-0.267 - 0.118) | |
| Exposure dur.: 9 yrs | | -0.116* | -0.259*** | -0.167*** | -0.183*** | | -0.212*** | -0.267*** | -0.260*** | -0.233** | |
|  | | (-0.242 - 0.009) | (-0.351 - -0.167) | (-0.276 - -0.057) | (-0.315 - -0.052) | | (-0.337 - -0.088) | (-0.366 - -0.167) | (-0.381 - -0.139) | (-0.432 - -0.034) | |
| Exposure dur.: 10 yrs | | -0.103 | -0.225*** | -0.089 | -0.174** | | -0.213** | -0.207*** | -0.248*** | -0.206** | |
|  | | (-0.289 - 0.083) | (-0.321 - -0.130) | (-0.208 - 0.030) | (-0.315 - -0.033) | | (-0.378 - -0.048) | (-0.315 - -0.099) | (-0.379 - -0.117) | (-0.411 - -0.002) | |
| Exposure dur.: 11 yrs | | -0.023 | -0.282*** | -0.249*** | -0.182** | | -0.127 | -0.196*** | -0.289*** | -0.257** | |
|  | | (-0.220 - 0.173) | (-0.399 - -0.164) | (-0.374 - -0.124) | (-0.353 - -0.012) | | (-0.298 - 0.045) | (-0.314 - -0.079) | (-0.440 - -0.139) | (-0.498 - -0.016) | |
| Exposure dur.: 12 yrs | | -0.034 | -0.258*** | -0.269*** | -0.206** | | -0.477*** | -0.295*** | -0.303*** | -0.293** | |
|  | | (-0.309 - 0.242) | (-0.389 - -0.128) | (-0.403 - -0.135) | (-0.386 - -0.025) | | (-0.671 - -0.283) | (-0.431 - -0.159) | (-0.482 - -0.125) | (-0.572 - -0.014) | |
| Exposure dur.: 14 yrs | | -0.406*** | -0.250*** | -0.281*** | -0.179** | | -0.339* | -0.262*** | -0.385*** | -0.264 | |
|  | | (-0.710 - -0.103) | (-0.405 - -0.095) | (-0.436 - -0.126) | (-0.347 - -0.011) | | (-0.696 - 0.018) | (-0.431 - -0.094) | (-0.598 - -0.172) | (-0.579 - 0.052) | |
| Exposure dur.: 14 yrs | | -0.535* | -0.291*** | -0.330*** | -0.030 | | -0.513** | -0.525*** | -0.313*** | -0.236 | |
|  | | (-1.072 - 0.002) | (-0.440 - -0.141) | (-0.521 - -0.138) | (-0.220 - 0.159) | | (-0.925 - -0.102) | (-0.710 - -0.341) | (-0.533 - -0.094) | (-0.613 - 0.141) | |
| Exposure dur.: 15 yrs | | -0.093 | -0.373*** | -0.141 | -0.252** | | -0.710 | -0.380*** | -0.355*** | -0.120 | |
|  | | (-0.327 - 0.141) | (-0.591 - -0.155) | (-0.335 - 0.054) | (-0.492 - -0.012) | | (-2.781 - 1.362) | (-0.582 - -0.177) | (-0.604 - -0.105) | (-0.462 - 0.221) | |
|  | |  |  |  |  | |  |  |  |  | |
| Observations | | 36,265 | 25,642 | 15,459 | 11,048 | | 36,279 | 20,336 | 10,482 | 4,955 | |

SRH - Self-rated Health; German Socio-Economic Panel 1994-2017; Significance levels are *p<0.05, **p<0.01 and ***p<0.001; Corresponds to Figure 5b

Shown are the average marginal effects (AME) for the specific exposure duration and the 95% confidence intervals as calculated from panel robust standard errors (SE)
